# Substitution of a Surface-Exposed Residue Involved in an Allosteric Network Enhances Tryptophan Synthase Function in Cells

**DOI:** 10.3389/fmolb.2021.679915

**Published:** 2021-05-26

**Authors:** Rebecca N. D’Amico, Yuliana K. Bosken, Kathleen F. O’Rourke, Alec M. Murray, Woudasie Admasu, Chia-en A. Chang, David D. Boehr

**Affiliations:** ^1^ Department of Chemistry, The Pennsylvania State University, University Park, PA, United States; ^2^ Department of Chemistry, The University of California Riverside, Riverside, CA, United States

**Keywords:** allostery, enzyme regulation, nuclear magnetic resonance, molecular dynamics, TIM barrel, substrate channeling, conformational dynamics

## Abstract

Networks of noncovalent amino acid interactions propagate allosteric signals throughout proteins. Tryptophan synthase (TS) is an allosterically controlled bienzyme in which the indole product of the alpha subunit (αTS) is transferred through a 25 Å hydrophobic tunnel to the active site of the beta subunit (βTS). Previous nuclear magnetic resonance and molecular dynamics simulations identified allosteric networks in αTS important for its function. We show here that substitution of a distant, surface-exposed network residue in αTS enhances tryptophan production, not by activating αTS function, but through dynamically controlling the opening of the indole channel and stimulating βTS activity. While stimulation is modest, the substitution also enhances cell growth in a tryptophan-auxotrophic strain of *Escherichia coli* compared to complementation with wild-type αTS, emphasizing the biological importance of the network. Surface-exposed networks provide new opportunities in allosteric drug design and protein engineering, and hint at potential information conduits through which the functions of a metabolon or even larger proteome might be coordinated and regulated.

## Introduction

Allosteric regulation of protein function is critical for several biological processes, including metabolism, oxygen transport and signal transduction (Wodak et al., 2019). Many enzymes are allosterically regulated, where a binding event distal to the active site impacts catalytic function (Monod et al., 1965; Koshland et al., 1966). Amino acid interaction networks have been proposed to connect different functional sites on a protein to propagate these allosteric signals (Dokholyan, 2016). Such networks might also bridge across proteins in multi-enzyme complexes, allowing efficient coordination of various enzyme functions (Cong et al., 2019; East et al., 2020). A better understanding of these protein-spanning networks would provide insight into how protein function is allosterically regulated in multi-enzyme complexes, and may offer novel avenues for protein engineering (Lee et al., 2008; Reynolds, et al., 2011; Gorman et al., 2019) and drug design (Nussinov and Chung, 2015; Greener and Sternberg, 2018).

**FIGURE 1:**
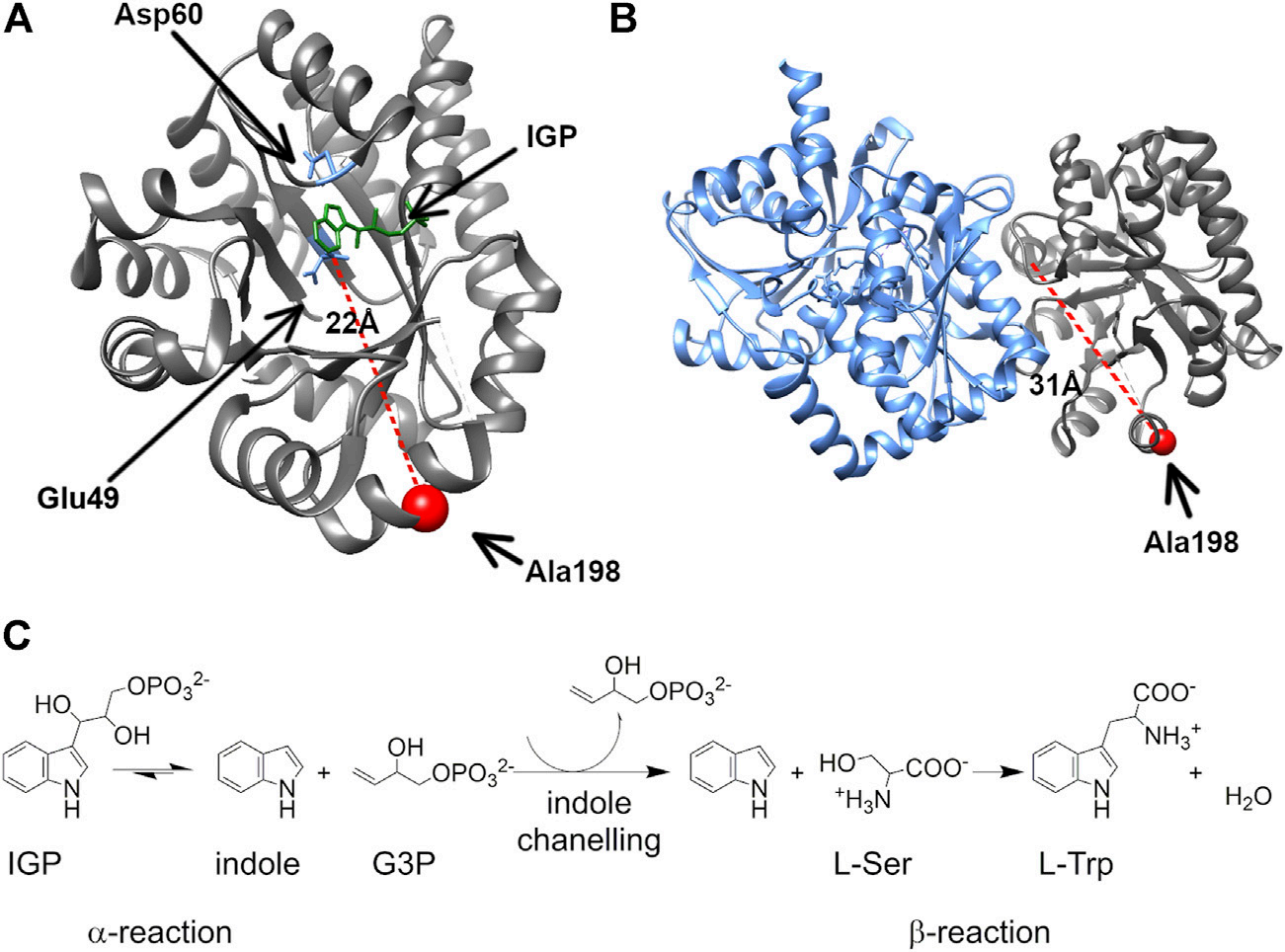
The surface-exposed, network residue Ala198 is remote from both the αTS active site and the αTS-βTS interface. Structures of **(A)** the αTS subunit (PDB ID Code: 2RH9), highlighting catalytic residues Glu49 and Asp60, the substrate IGP, and Ala198, and **(B)** the full tryptophan synthase complex with the βTS subunit pictured in blue **(C)** Reactions catalyzed by alpha and beta subunits of TS.

TS has emerged as a model system for understanding allosteric regulation and functional coordination in multi-enzyme complexes (Dunn, 2012). TS contains both αTS and βTS subunits in a linear αββα arrangement (Figure 1). The TS enzyme is an anti-microbial drug target in *Mycobacterium tuberculosis* (Abrahams et al., 2017; Wellington et al., 2017) and has been implicated in antibiotic resistant strains of *Chlamydia tracomatis* (Somboonna et al., 2019). There has also been interest in engineering βTS for the production of novel tryptophan analogs (Buller et al., 2015; Murciano-Calles et al., 2016; Buller et al., 2018). TS has been of particular interest due to the channeling of the intermediate, indole, between the two subunits through a 25 Å hydrophobic tunnel (Dunn et al., 1990). Structural changes in TS appear to be highly coordinated as structural changes in one subunit affects the structure and catalytic activity of the other subunit (Dunn, 2012). Notably, both subunits experience a marked decrease in catalytic activity in the absence of the other subunit (Niks et al., 2013). Understanding how structural changes and function are coordinated between the TS subunits may provide insight into similar interactions in other multi-subunit enzyme complexes.

We previously delineated amino acid interaction networks for αTS in the absence of βTS (Axe et al., 2014; O’Rourke et al., 2019) using the solution-state nuclear magnetic resonance (NMR) method known as CHESCA (chemical shift covariance analysis) (Selvaratnam et al., 2011). Interestingly, these networks included residues at the αTS/βTS binding interface, which suggested that they might be involved in coordinating function between αTS and βTS. A particularly interesting residue was Ala198, a surface-exposed network residue that is conformationally dynamic throughout the catalytic cycle of αTS (O’Rourke et al., 2018), and is distant from both the αTS active site and the αTS/βTS binding interface. Previous studies indicated that the A198G substitution induces a modest decrease in αTS catalytic activity, and changed the structure/dynamics of other network residues, according to NMR studies (Axe et al., 2014). Here, we have developed a cell-based assay to screen the phenotypic consequences of network substitutions of αTS, in particular, evaluating network substitutions at position 198. Surprisingly, we show that the A198W substitution induces more rapid bacterial growth in a tryptophan auxotrophic strain of *Escherichia coli*, likely by increasing catalytic efficiency of TS. Several computational approaches have been developed in an effort to map allosteric networks which commonly rely on tracing significant changes in residues conformation and interaction (Feher et al., 2014; Johnson et al., 2018; Botello-Smith and Luo, 2019; Wang et al., 2020). Similarly, in this study we evaluated pairwise forces to distinguish specific interaction changes resulting from mutation. The A198W substitution induces structural and dynamic changes to the allosteric network that connects to the αTS/βTS interface resulting in new connections, opening of the indole channel and more efficient tryptophan biosynthesis. These studies underscore the importance of network residues not only to enzyme function but to coordination of functions between enzymes.

## Materials and Methods

### *Escherichia coli* Growth Curves

Growth curves were determined using *E* ***.*** *coli* K-12 BW25113 cells with the trpA gene knocked out. Cells were provided by the Keio Collection at Yale University (Baba et al., 2006). The kanamycin cassette was then eliminated using the lambda red recombinase method (Datsenko and Wanner, 2000). Cells were grown for 16 h in Vogel-Bonner minimal media supplemented with 1X MEM vitamin mix, and 7.5 µM tryptophan. Cell lines containing a trpA plasmid were also grown in the presence of 50 μg/ml kanamycin. After 16 h, the resulting cultures were diluted 1:10 in identical medium containing 7.5 µM tryptophan and were grown for 8 h. Cell cultures were then diluted 1:25 in identical medium lacking tryptophan. Growth over a period of 50–60 h was measured using OD_600_ readings. Resulting curves were plotted and fit using the following equation:

$$N_{t}=\;\frac{K}{1+\left(\frac{K-N_{0}}{N_{0}}\right)e^{-rt}}$$

where the *N*_0_ represents population at time zero and *N*_*t*_ represents the population size at time *t*. *K* represents the carrying capacity–or the maximum population size that could be supported with no limitations. This equation was adapted from the Growthcurver package (Sprouffske and Wagner, 2016) for use in MatLab (MathWorks, Natick, MA, United States). Error for each fit was calculated using residual bootstrapping with 1,000 repetitions.

### Generation of Position 198 Variants

The A198C, A198F, A198G, A198N, A198Q, and A198W variants were obtained using QuikChange Lightning Site Directed Mutagenesis Kit (Agilent Technologies). Primers were designed by using Agilent’s primer design tool. The remaining variants (A198D, A198V, A198R, A198L, A198K) were obtained using the Q5 Site-Directed Mutagenesis Kit (New England Biolabs, NEB) using a site-saturation method. These primers were designed using NEB’s supplied primer design tool.

### Expression and Purification of αTS and βTS

All proteins were expressed using *E. coli* BL21 (DE3*) cells. Expression of unlabeled proteins was carried out using Luria-Bertrani (EMD Millipore, Billerica, MA, United States) medium, and expression of isotopically labeled proteins was carried out using ^2^H_2_O-based M9 medium with ^15^NH_4_Cl by previously described methods (Axe and Boehr, 2013; Axe et al., 2014). The expressed βTS subunit contained a N-terminal hexahistidine tag.

The αTS subunit and variants were purified as previously (Axe and Boehr, 2013; Axe et al., 2014; O’Rourke et al., 2019). The βTS subunit was purified using a Ni-NTA resin using column buffer (25 mM HEPES, pH 7.5, 1 mM Na_2_EDTA, 200 mM NaCl) and a gradient of imidazole from 50–500 mM. The βTS subunit eluted at 250 mM imidazole. Samples were then concentrated using a Corning Spin-X UF centrifugal concentrator and applied to a size exclusion S200 column (GE Healthcare) and eluted with column buffer.

### Enzyme Kinetic Assays

The chemoenzymatic production of indole-3-glycerol phosphate (IGP) substrate was carried out as previously described (O’Rourke et al., 2019). For enzyme assays, the IGP concentration was determined using *ε* = 5.54 mM^−1^ cm^−1^. Assays of the αTS subunit alone were performed using a previously described assay (Creighton, 1970; Lane and Kirschner, 1991), which utilizes a coupled reaction scheme as follows:

$$\text{IGP}\;\overset{\alpha TS}{\to }\text{Indole}+\text{G}3\text{P}$$

$$\text{G}3\text{P}+{\text{NAD}}^{+}+{\text{AsO}}_{4}^{3-}\overset{\text{GAPDH}}{\to }\text{NADH}+{\text{H}}^{+}+\text{BPG}$$

where G3P is glyceraldehyde-3-phosphate, NAD^+^ and NADH are the oxidized and reduced forms of nicotinamide adenine dinucleotide, and BPG is 1,3 bisphosphoglycerate. Production of NADH was measured at 340 nm using *ε* = 6.22 mM^−1^ cm^−1^. The assay was performed in assay buffer (100 mM HEPES pH 7.5, 100 mM KCl, and 40 µM pyridoxal 5′ phosphate) plus 20 mM sodium arsenate. The concentration of the αTS subunit was 60 µM and the IGP concentration was 0.075–2.25 mM.

The catalytic activity of the βTS subunit (Faeder and Hammes, 1970) was assayed in the presence of wild-type αTS or variants by measuring the production of tryptophan at 290 nm using *ε* = 1.89 mM^−1^ cm^−1^. The concentration of the α_2_β_2_ TS complex was 0.5 µM. The reaction was initiated using 1–50 µM indole (Thermo Fisher). Assays were performed in the same assay buffer as indicated above, plus 60 mM serine.

The full TS complex reaction (Brzovic et al., 1992) was measured by tracking production of tryptophan from IGP at 290 nm using ε = 1.89 mM^−1^ cm^−1^. The reaction was initiated with 0.05–3 mM of IGP. The concentration of the α_2_β_2_ TS complex used was 50 µM and assays were performed in assay buffer, plus 60 mM serine.

Given that some of the kinetic data was collected under conditions in which the total substrate concentration [(S_T_)] approached the total enzyme concentration ([E_T_]), the kinetic data was fit to the Morrison quadratic equation (Morrison, 1969):

$$v_{0}=V_{\max}\frac{\left(\left[E_{T}\right]+\left[S_{T}\right]+K_{M}\right)-\sqrt{{\left(\left[E_{T}\right]+\left[S_{T}\right]+K_{M}\right)}^{2}-4\left[E_{T}\right]\left[S_{T}\right]}}{2\left[E_{T}\right]}$$

where *v*_o_ is the initial reaction velocity, *V*_max_ is the maximum reaction velocity and *K*_*M*_ is equivalent to the Michaelis–Menten constant. The maximum catalytic rate constant (*k*_cat_) is determined by dividing *V*_max_ by [*E*_*T*_] as normal.

For each initial velocity vs. [*S*] curve, kinetic data was collected for eight different [*S*] data points, in which these data points represent three technical replicates (i.e., using the same batch of purified enzyme), and each curve was generated in totality at least three times with different biological replicates (i.e., using different batches of purified enzyme).

### NMR Experiments

The NMR experiments were conducted and analyzed as previously described (Axe and Boehr, 2013; O’Rourke et al., 2018). Protein samples were exchanged into NMR buffer (50 mM potassium phosphate, pH 7.8, 2 mM DTT, 0.2 mM EDTA, and 10% ^2^H_2_O) and contained 1.0 mM ^2^H, ^15^N-labeled αTS, 10 mM indole (Thermo Fisher) and/or 20 mM G3P (Sigma Aldrich) where appropriate. ^15^N R_2_ relaxation dispersion experiments were collected and analyzed according to previously established procedures (Loria et al., 2008; O’Rourke et al., 2018). Briefly, data was collected at 283 K on 600 and 850 MHz Bruker Avance III spectrometers using previously described pulse sequences (Loria et al., 1999) and data analyzed using the computer program GLOVE (Sugase et al., 2013).

### Molecular Dynamics Simulations and Analysis

Due to the lack of X-ray crystal structure for the *E. coli* α_2_β_2_ TS complex, wild type and A198W mutant molecular systems were based on the X-ray crystal structure of *Salmonella typhimurium* TS PDB ID 2CLK (Ngo, Harris et al., 2007) and corresponding residues were substituted to match the *E. coli* sequence. The coordinates for the active conformation of Glu49 and the substrates were taken from PDB entry 1QOQ (Weyand and Schlichting, 2000).

Four independent simulations were performed for each system (different random seed, starting from the same equilibrated system)—wild type (WT) and variant (A198W). MD simulations were performed using standard Amber package with GPU acceleration (Salomon-Ferrer et al., 2013; Case et al., 2018). The protein was parameterized using Amber Force Field FF14SB (Maier et al., 2015). General Amber force field (GAFF) was applied to ligands and charges were assigned using AM1-bcc model (Jakalian et al., 2002). All systems were prepared by a three-step minimization process (hydrogens, sidechains and all atoms), solvated with TIP3P water model with counter ions in a rectangular box with edges at minimum 12 Ǻ from any atom (Jorgensen et al., 1983). The solvated systems were minimized and equilibrated from 0 to 298 K at 25 K intervals. MD trajectories were collected over 200 ns at 1 ps interval with 2 fs timestep under constant pressure and temperature. Particle mesh Ewald was used for long range electrostatics and SHAKE algorithm for fixed heavy atom–hydrogen bond lengths (Ryckaert et al., 1977; Sagui et al., 2004).

The systems were visualized and analyzed using Visual Molecular Dynamics (Humphrey et al., 1996) and Molecular Operating Environment. The trajectory output files were processed with PTRAJ software to contain 2,000 frames, each representing 0.1 ns timestep. Dihedral data was collected with T-Analyst software (Ai et al., 2010; Roe and Cheatham, 2013). Dihedral entropy was also calculated using T-Analyst and residues with difference in entropy between WT and variant higher than 0.2 kcal/mol were selected for further analysis (sidechain and backbone dihedral angles were considered). Four MD runs were analyzed for the WT and A198W variant systems. The force distribution analysis tool was used to identify significant contacts and persistent interactions throughout the 200 ns trajectories. The pair wise atom forces are represented as a scalar value with negative values indicating attraction and positive values showing repulsion (Stacklies et al., 2011).

## Results

### The A198W Network Substitution Engenders a Fast Growth Phenotype to *E.coli* Cells

We had previously identified network substitutions that are modestly detrimental to the kinetic parameters of αTS (Axe et al., 2014). We sought to develop a cell growth-based assay that would allow us to analyze αTS substitutions in a much higher throughput manner, and especially evaluate any phenotypic responses to network substitutions. Towards this goal, we obtained *E. coli* K12 cells with the trpA gene knockout from the Keio collection at Yale University (Baba et al., 2006), and analyzed growth in the presence and absence of tryptophan, with and without complementing plasmid expressing *E. coli* αTS (Supplementary Figure S1). As expected, cells that were not supplemented with tryptophan and did not carry the αTS-expressing plasmid failed to grow. Interestingly, the cells supplemented with plasmid but not tryptophan experienced a delay in the start of the growth phase. This phenomenon has been previously observed in *E. coli* K12 strains, including isoleucine and serine auxotrophs (Fisher et al., 2011). Fisher and coworkers were able to rescue auxotroph viability with *de novo* proteins for several amino acid auxotrophs. The authors attributed the slower cell growth to the *de novo* proteins being less efficient than the endogenous protein. However, we observed this lag even when the cells were supplemented with the wild-type (WT) version of the knocked-out enzyme. We attributed this lag to the additional metabolic stress induced by the required plasmid. This lag was observed consistently in cells that required the plasmid to produce tryptophan for cell growth.

**FIGURE 2:**
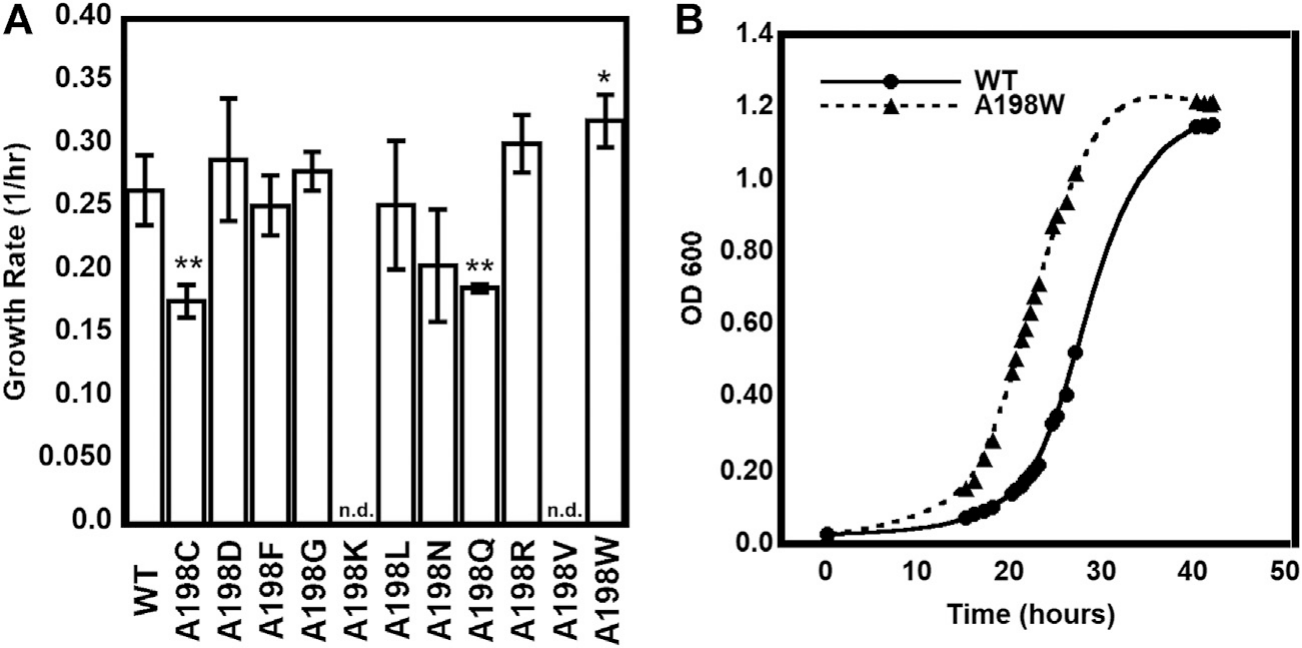
Growth complementation assays of trpA-deleted *Escherichia coli* K12 cells. **(A)** Tryptophan-auxotrophic *E. coli* cells were complemented with plasmid expressing αTS WT or A198 variants, and growth rates determined. Asterisks indicate that the growth rate for *E. coli* expressing the A198 variant was statistically different than the growth rate for *E. coli* expressing WT αTS, according to *p* value (0.01 < * < 0.05; 0.001 < ** < 0.01; *n* = 3). n. d. not determined because *E. coli* cells expressing the A198K and A198V variants did not grow in the absence of tryptophan **(B)** Representative growth curves demonstrating the A198W variant’s increased growth rate.

We were especially interested in evaluating substitutions at position 198 of αTS. Ala198 is an allosteric network residue (Axe et al., 2014; O’Rourke et al., 2019) that is distant from both the αTS active site (>20 Å) and the αTS/βTS binding interface (>30 Å) (Figure 1). Nonetheless, a substitution at position 198 has a modest effect on the αTS kinetic parameters (Axe et al., 2014). Moreover, Ala198 has been shown by NMR ^15^N R_2_ relaxation dispersion experiments to be conformationally dynamic on the millisecond timescale in the presence and absence of substrate/products (O’Rourke et al., 2018). In order to investigate the structural and/or functional relevance (if any) of Ala198, especially within a biologically relevant context, we generated several point mutations at this position and tested each of these point variants using the *E. coli* K12 knockout cells. We found that several amino acid substitutions caused a statistically significant decrease in cellular growth rate (i.e., A198C and A198Q) while others were not able to rescue growth (i.e., A198K and A198V) (Figure 2A). Most surprisingly, production of the A198W variant resulted in an increase in cellular growth rate (Figure 2B). These results indicated that position 198 was indeed structurally/functionally important for TS. We note that expression levels of WT and A198W αTS were very similar (Supplementary Figure S2), suggesting that cell growth differences were likely related to functional differences induced by the A198 substitutions. As it is atypical for amino acid substitutions to enhance enzyme function, most of the remaining studies focused on understanding the functional, structural and dynamic consequences of the A198W substitution. We note first that we repeated the growth curve experiments for WT (*n* = 13) and A198W (*n* = 9) to determine the extent of this increased rate. The average growth rate for cells expressing the A198W variant (0.3375 ± 0.0400) was consistently higher than that for cells expressing WT αTS (0.2664 ± 0.0549; *p* = 0.0035).

### The A198W Substitution Increases Catalytic Efficiency of The Full TS Complex

To better understand the effects of the A198 substitutions on the structure and function of the TS enzyme, we expressed and purified the A198K, A198V, and A198W variants. The A198K and A198V NMR spectra were very different from that of WT αTS (Supplementary Figure 3), including many chemical shift and peak intensity changes. Unfortunately, these changes precluded ready NMR assignments for these spectra. We note in the next section that the A198W variant also led to changes in the NMR spectrum, but these changes were not nearly as severe as those induced by the A198K and A198V variants (Supplementary Figure 3). Given these results, the remaining studies were performed only with the A198W variant.

We conducted a number of kinetic experiments to better elucidate the effects of the A198W substitution on the function of the TS enzyme (Table 1, Supplementary Figure 4). We note that some of the substrate concentrations approached the total enzyme concentration. In these cases, it is better to model the steady-state kinetics using the Morrison quadratic equation (Morrison, 1969), which also provides the Michaelis–Menten constant (*K*_*M*_) and the catalytic turnover rate constant (*k*_cat_). However, the kinetic parameters determined by the Morrison and Michaelis–Menten equations were nearly identical and not statistically different. We report here only the kinetic parameters determined through the Morrison equation for simplification.

**TABLE 1:** Kinetic parameters for WT and A198W TS.

Assay	Protein	*k*_cat_ (s^−1^)	*K*_*M*,IGP_ (mM)	*K*_*M*,indole_ (μM)	*k*_cat_/*K*_*M*_ (M^−1^s^−1^)	(*k*_cat_/*K*_*M*_)^A198W^/(*k*_cat_/*K*_*M*_)^WT^
αTS alone	WT	(2.88 ± 0.54) × 10^–4^	0.94 ± 0.28		0.31 ± 0.11	
	A198W	(3.81 ± 10.16) × 10^–4^	1.31 ± 0.09		0.29 ± 0.02	0.94 ± 0.34
TS complex	WT	2.83 ± 0.24	0.49 ± 0.10		(5.8 ± 1.2) × 10^3^	
	A198W	4.14 ± 0.25	0.42 ± 0.06		(9.9 ± 1.4) × 10^3^	1.7 ± 0.4
βTS assay	WT	3.81 ± 0.38		25 ± 5	(1.5 ± 0.3) × 10^5^	-
	A198W	3.08 ± 0.26		16 ± 4	(1.9 ± 0.5) × 10^5^	1.3 ± 0.4

We first analyzed the effects of the A198W substitution on the catalytic activity of αTS in the absence of βTS. The activity of αTS alone was measured via a coupled reaction (Lane and Kirschner, 1991), in which production of product G3P from the αTS forward reaction was coupled to reduction of NAD^+^, generating a measurable absorbance change at 340 nm. Given the results of the cell-based assay, it was surprising that there did not appear to be any significant difference for the catalytic efficiencies (*k*_cat_/*K*_*M*_) between WT (0.31 M^−1^ s^−1^) and A198W (0.29 M^−1^ s^−1^) αTS.

We then compared the kinetic parameters of the entire WT and A198W TS complexes by measuring the production of tryptophan from IGP at 290 nm. Tryptophan production requires reactions to occur in both subunits, with the product of the αTS reaction (indole) being channeled to the βTS subunit. In contrast to the αTS assays, there was a modest increase in the catalytic efficiency of the A198W TS variant (9.9 × 10^3^ M^−1^ s^−1^) compared to WT TS (5.8 × 10^3^ M^−1^ s^−1^). We also tested the ability of the A198W αTS subunit to stimulate βTS activity by measuring the production of tryptophan starting from indole at 290 nm. Here, there was also a slight increase in the catalytic efficiency in the presence of A198W αTS (A198W, 1.9 × 10^5^ M^−1^ s^−1^; WT, 1.5 × 10^5^ M^−1^ s^−1^), although this appeared to be driven by a small decrease in the apparent *K*_*M*_, whereas the change in the catalytic efficiency for the full TS complex assay was driven primarily by an increase in k_cat_ in the presence of A198W αTS (see Table 1).

While the kinetic effects of the A198W substitution were admittedly modest (1.4–1.7 fold), these effects were on par with the faster growth rate (∼1.3 fold) in the *E. coli* cells expressing A198W αTS. For comparisons, we reference some studies by the Arnold lab in trying to engineer stand-alone βTS enzymes for chemoenzymatic processes. As indicated, αTS and βTS have decreased enzyme activity in the absence of the other subunit (Niks et al., 2013). However, the Arnold lab was able to use directed evolution on the *Pyrococcus furiosus* βTS to identify βTS enzymes with higher catalytic activities, comparable to those found with the full TS complex (Buller et al., 2015). A recombination library of these original mutations enabled the discovery of mutations that also enhanced the catalytic activity of the isolated *Thermotoga maritima* βTS, such that the engineered βTS had catalytic activities ∼2.5–4.5 greater than that of the full TS complex (Murciano-Calles et al., 2016). However, it is notable that similar strategies failed to identify *E. coli* βTS variants with enhanced activity (Murciano-Calles et al., 2016).

### The A198W Substitution Does not Affect Binding Affinity Between the αTS and βTS Subunits

One potential explanation for the increased activity of the TS complex was that the A198W substitution could be impacting the binding affinity between αTS and βTS. To investigate this, isothermal titration calorimetry (ITC) studies were performed on the WT and the A198W variant αTS subunit binding to the βTS subunit (Supplementary Figure 5). WT and A198W αTS bound βTS with nearly identical nanomolar dissociation constants (∼25 nM). These experiments showed that there was no significant thermodynamic difference between the two binding events, implying that functional changes due to the A198W substitution were not simply due to differences in subunit association thermodynamics.

### The A198W Substitution Attenuates Conformational Exchange Events Including at the αTS/βTS Interface

**FIGURE 3:**
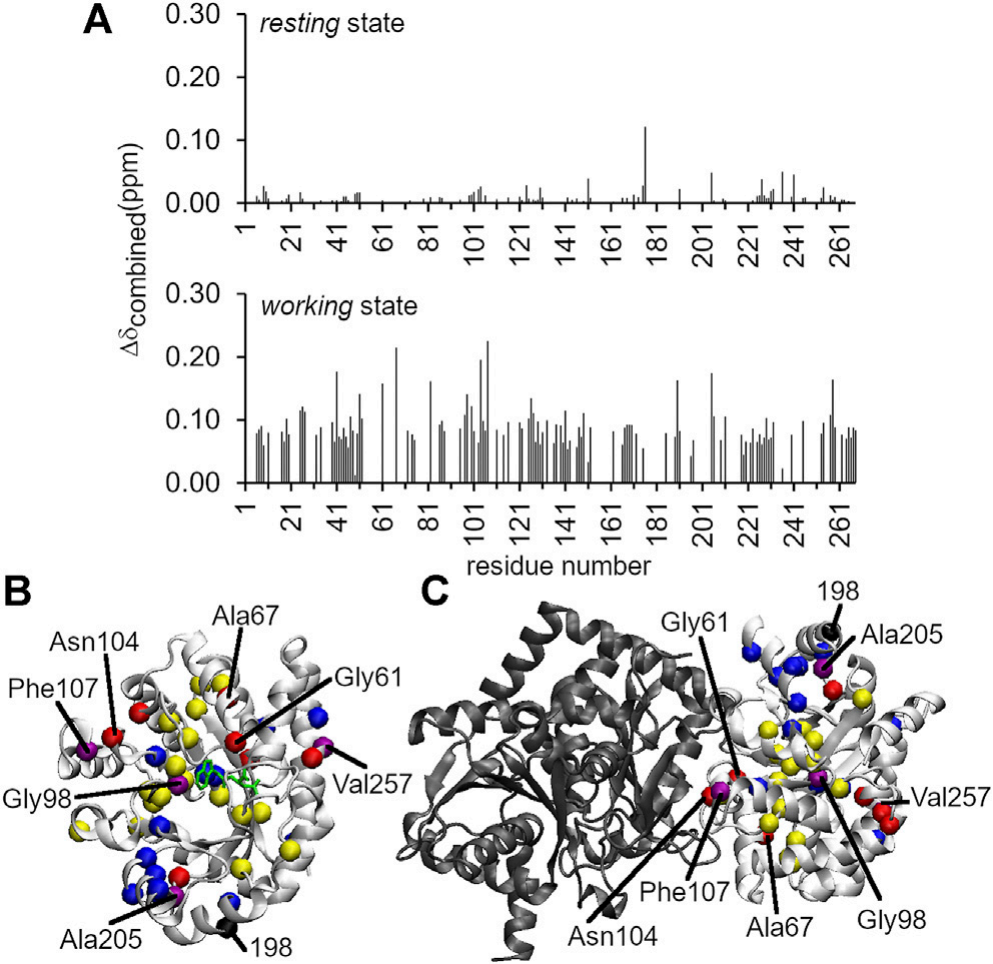
Long-range structural and dynamic changes induced by the A198W substitution. **(A)** ^1^H–^15^N chemical shift perturbations induced by the A198W substitution. The Δ*δ*_combined_ values were determined according to the equation, (Δ*δ*_combined_ = (Δ*δ*_HN_^2^ + (Δ*δ*_N_/5)^2^)^0.5^, where Δ*δ*_HN_ and Δ*δ*_N_ were the ^1^H and ^15^N amide chemical shift differences between WT and A198W. Chemical shift differences between WT and A198W αTS are shown for both the ligand-free *resting* state (top; average Δ*δ*_combined_ < 0.01 ppm), and the actively turning over *working* state (bottom; average Δ*δ*_combined_ = 0.04 ppm). Residues with Δ*δ*_combined_ values greater than 0.15 ppm and between 0.10 and 0.15 ppm for the *working* state are plotted as red and yellow spheres, respectively, onto the structures of **(B)** αTS and **(C)** the full TS complex. Network residues are plotted as blue spheres, or as purple spheres if they met the Δ*δ*_combined_ thresholds (i.e., > 0.10 ppm). Most of the network residues showed chemical shift perturbations but may not have met this threshold. It should be noted that NMR experiments were performed in the absence of βTS (shown in black).

**FIGURE 4:**
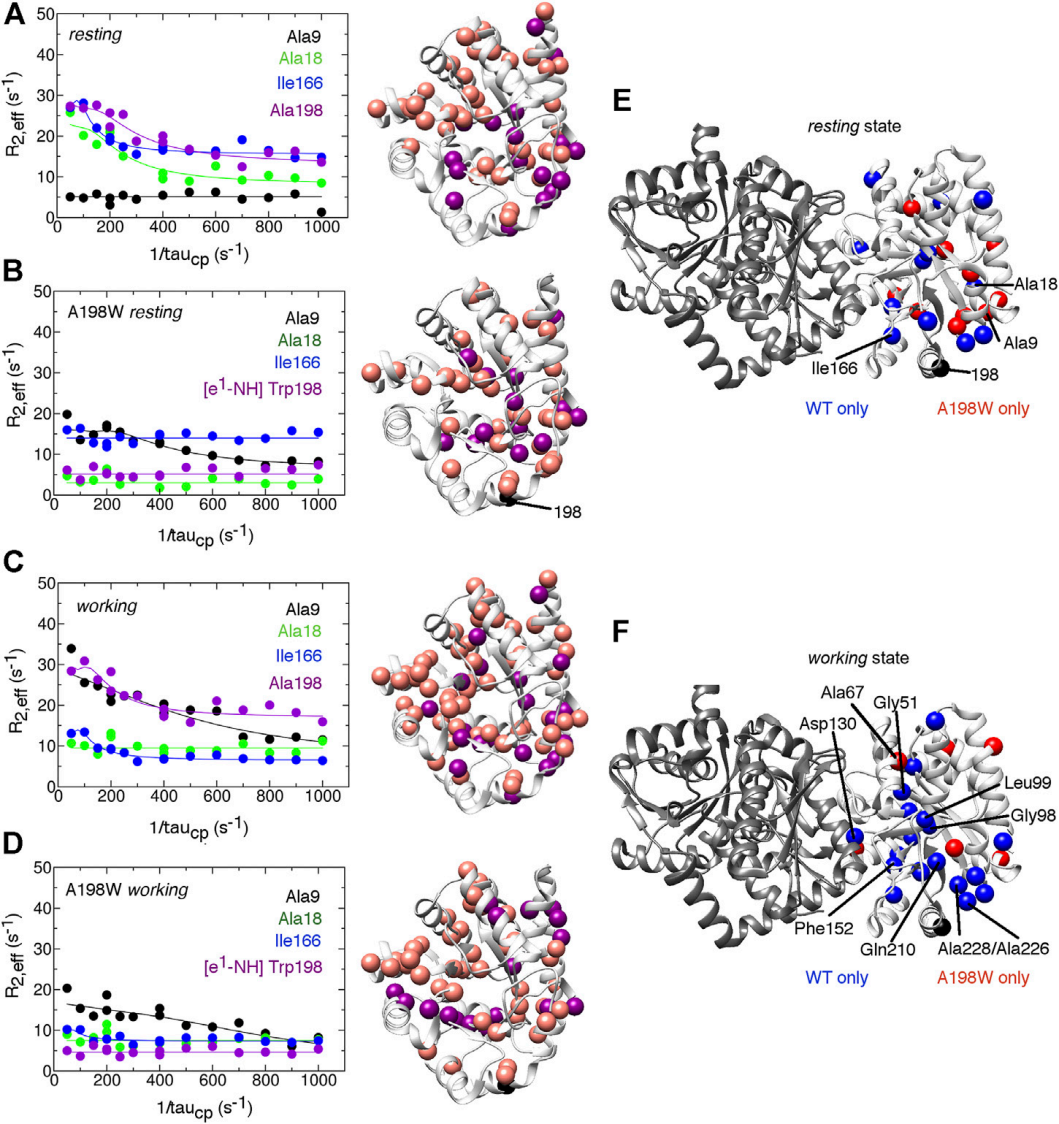
The A198W surface-exposed, network substitution suppresses millisecond conformational motions in αTS. Conformational exchange events in *E. coli* αTS enzyme for **(A)** WT ligand-free *resting*, **(B)** A198W ligand-free *resting*, **(C)** WT *working* and **(D)** A198W *working* states. **(Left)** Example ^15^N R_2_ relaxation dispersion curves collected at a ^1^H Larmor frequency of 850 MHz for resonances belonging to Ala9 (black), Ala18 (green), Ile166 (blue), and the sidechain ε^1^-NH group of Trp198 (purple). **(Right)** Locations of conformational exchange according to the R_2_ relaxation dispersion curves plotted as spheres onto the αTS structure. Purple spheres indicate that associated R_2_ relaxation dispersion curves can be fit to two-site exchange, while pink spheres indicate exchange broadening, but the R_2_ relaxation dispersion curves cannot be fit reliably to two-site exchange. Comparison of the conformational exchange events between WT and A198W αTS for **(E)** *resting* and **(F)** *working* states is also shown. Blue (red) spheres indicate conformational exchange events present in the WT (A198W) enzyme but not in the A198W (WT) enzyme. It should be noted that the NMR data was collected in the absence of βTS. The ^15^N R_2_ relaxation dispersion experiments were conducted at 283 K using a buffer consisting of 50 mM potassium phosphate, pH 7.8, 2 mM DTT, 0.2 mM Na_2_EDTA, and 10% ^2^H_2_O, and 0.5–1.0 mM protein with 10 mM indole, and/or 20 mM G3P, where appropriate.

Considering that we had previously identified Ala198 as a potentially important residue by NMR (Axe et al., 2014; O’Rourke et al., 2018; O’Rourke et al., 2019), we also evaluated structure/dynamic changes induced by A198W through NMR methods. We note that these NMR experiments were conducted in the absence of βTS. We collected ^1^H–^15^N HSQC spectra (Figure 3) and ^15^N R_2_ relaxation dispersion (Figure 4) data for the ligand-free *resting* state and the *working* state of αTS. The working state represents active turnover conditions to reach dynamic conformational equilibrium between the E:IGP and E:indole:G3P states in a 4:1 ratio (Axe and Boehr, 2013).

Intriguingly, the chemical shift perturbations induced by the A198W substitution were more substantial in the *working* state than in the ligand-free *resting* state. The average chemical shift perturbations for the *resting* and *working* state conditions were < 0.01 and ∼0.04 ppm, respectively {as measured by Δ*δ*_total_ = [Δ*δ*_HN_^2^ + (Δ*δ*_N_/5)^2^]^0.5^, where Δ*δ*_HN_ and Δ*δ*_N_ were the ^1^H and ^15^N amide chemical shift differences between WT and A198W αTS}. In the *resting* state, Leu176 experienced the largest chemical shift perturbation with Δ*δ*_total_ = 0.12 ppm (Figure 3). In the *working* state, the A198W substitution induced chemical shift perturbations throughout αTS (Figure 3), with the largest chemical shift perturbations associated with the β2α2 active site loop (i.e., Ala67) and residues at the αTS/βTS interface (i.e., Asn104, Phe107). Our previous NMR studies (Axe et al., 2014; O’Rourke et al., 2019) identified two network clusters, and most residues experiencing substantial chemical shift perturbations (i.e., Δ*δ*_total_ > 0.10 ppm) belonged to one of these clusters, or were next to a network residue.

The A198W substitution also led to changes in millisecond conformational exchange events in both the *resting* and *working* states of αTS according to the ^15^N R_2_ relaxation dispersion experiments (Figure 4). In the *working* state, the A198W substitution mostly suppressed protein motions that were present in the WT enzyme. These suppressed motions included those near the substitution site (e.g., Ala226, Ala228), near the active site (e.g., Gly51, Gly98, Leu99, Phe152, Gln210) and near the αTS/βTS interface (e.g., Asp130). The rate of conformational exchange also slowed for those residues still undergoing exchange in A198W αTS (Supplementary Table 1). Together the chemical shift perturbations and ^15^N R_2_ relaxation dispersion data suggest that there are dynamic connections between position 198, the active site and the αTS/βTS interface, thus providing potential means through which substitutions at position 198 can influence TS function. Conversely, it is remarkable that despite all of these dynamic changes, there is little effect on the kinetic parameters of αTS (Table 1). The function of αTS in isolation may be robust to these dynamic changes, although it is noted that αTS acts as a very poor catalyst in the absence of βTS.

#### Bioinformatics Analyses of the NMR-Derived Allosteric Networks

The A198W substitution appeared to influence TS function by modulating interactions between αTS and βTS, especially changing interactions directly involving or nearby previously identified allosteric network residues. Other biophysical and computational methods exist that can also help to delineate allosteric networks (O’Rourke et al., 2016). For example, the concept of “frustration” can provide insight into how energy is distributed in protein structures and how mutations or conformational changes shift these energy distributions (Ferreiro et al., 2007). In the algorithm developed by Ferreiro et al. (2007), local frustration is determined by mutating single residues or pairs of residues *in silico* and computing energy changes. If the native residue or pair is destabilizing compared to alternatives, this residue or pair interaction is “frustrated”. Sites with high frustration are often associated with binding or allosteric sites, and may be important for guiding functionally-relevant dynamics (Ferreiroet al., 2014; Ferreiro et al., 2018; Freiberger et al., 2019). We used the AWSEM-MD Frustratometer (Parra et al., 2016) to identify highly frustrated residues in the related *Salmonella typhimurium* αTS, which has 85% sequence identity to *E. coli* αTS. We evaluated frustration for a number of complexes, including TS bound with the αTS substrate mimic N-[1H-indol-3-yl-acetyl] aspartic acid (PDB 1K3U), TS bound with the αTS transition state analog 4-(2-hydroxy-4-fluorophenylthio)-butylphosphonic acid (PDB 1C9D), TS bound with αTS inhibitor F9 and L-tryptophan in the beta site (PDB 5CGQ) and TS bound with the βTS quinoid intermediate (PDB 3CEP). The most highly frustrated residues were similar for the different TS structures. Specifically, residues Glu2, Pro28, Gln32, Asp46, Asp56, Asn66, Glu83 Asp130, Glu135, and Lys263 (all conserved between *E. coli* and *S. typhimurium* αTS) were identified as highly frustrated in all evaluated structures (Supplementary Figure 6). It is noteworthy that Gln32, Asp46, Asp130, and Lys263 were all identified as network residues according to the previous NMR studies (O’Rourke et al., 2019); the backbone resonances for Glu2 and Asn66 were unassigned, the ^1^H–^15^N based NMR experiments could not provide information about Pro28 although Asp27 was a network residue, and Ser136 (i.e., next to Glu135) was also identified as a network residue. The A198W substitution induced ^1^H–^15^N backbone amide chemical shift changes (e.g., Asp27, Ala47) and/or motional changes (e.g., Asp130, Val131) to some of these or nearby residues.

Another highly used method to identify allosteric networks in proteins involves identifying covarying or coevolving residue pairs. In this method, statistical analyses are performed on large multiple sequence alignments to identify if two residues covary/coevolve across the alignment (dos Santos et al., 2019; Morcos and Onuchic, 2019). Residues that covary likely interact, and may be involved in the same allosteric network (Rivoire et al., 2016). For our purposes, we used the RaptorX-Complex Contact webserver (Zeng et al., 2018) to identify covarying residues within αTS and between αTS and βTS using the *E. coli* sequences as starting points. We identified the top ten covarying pairs of residues within αTS (see Supplementary Figure 6). For seven of these pairs, one or both residues are NMR-derived network residues (O’Rourke et al., 2019). The other three pairs contain a residue that is next to a network residue (e.g., covarying residue Ser125 in two of the pairs is next to Asp124; covarying residue Ala265 is between the network residues Ala264 and Thr266). We also identified the top covarying pairs of residues between αTS and βTS (see Supplementary Figure 6). Perhaps not surprisingly, and likely owing to the power of the algorithm, all of these pairs are at the αTS-βTS interface. These residues include those previously identified as network residues (i.e., Ala103, Asn104 and Phe107) (O’Rourke et al., 2019). The A198W substitution induces ^1^H–^15^N backbone amide chemical shift changes (e.g., Ala47, Gly51, Gly98, Leu100, Asn104, Phe107) and/or motional changes (e.g., Gly51, Gly98, Asp124, Val148, Gln210, Ala231, Ala265) to many of these covarying residues or nearby residues in αTS.

#### Molecular Dynamics Simulations Indicate that the A198W Substitution Induces a More Open Indole Channel

While the NMR data provided some insight into communication between position 198 and the αTS/βTS interface, the NMR data was limited in providing full context for the αββα TS complex. As such, we analyzed molecular dynamics (MD) trajectories for α−β heterodimers, including for WT, A198K, A198V, and A198W αTS. The MD simulations for the A198K and A198V variants indicated a dramatic movement of active site loop 6 (also known as the β6α6 loop or as αL6) into a position that completely opens the active site and which is likely not conducive for catalysis (Supplementary Figure 7); this αL6 conformation was not observed for either WT or A198W αTS. The large conformational change in αL6 may help to explain the large chemical shift and peak intensity changes in the A198K and A198V NMR spectra.

**FIGURE 5:**
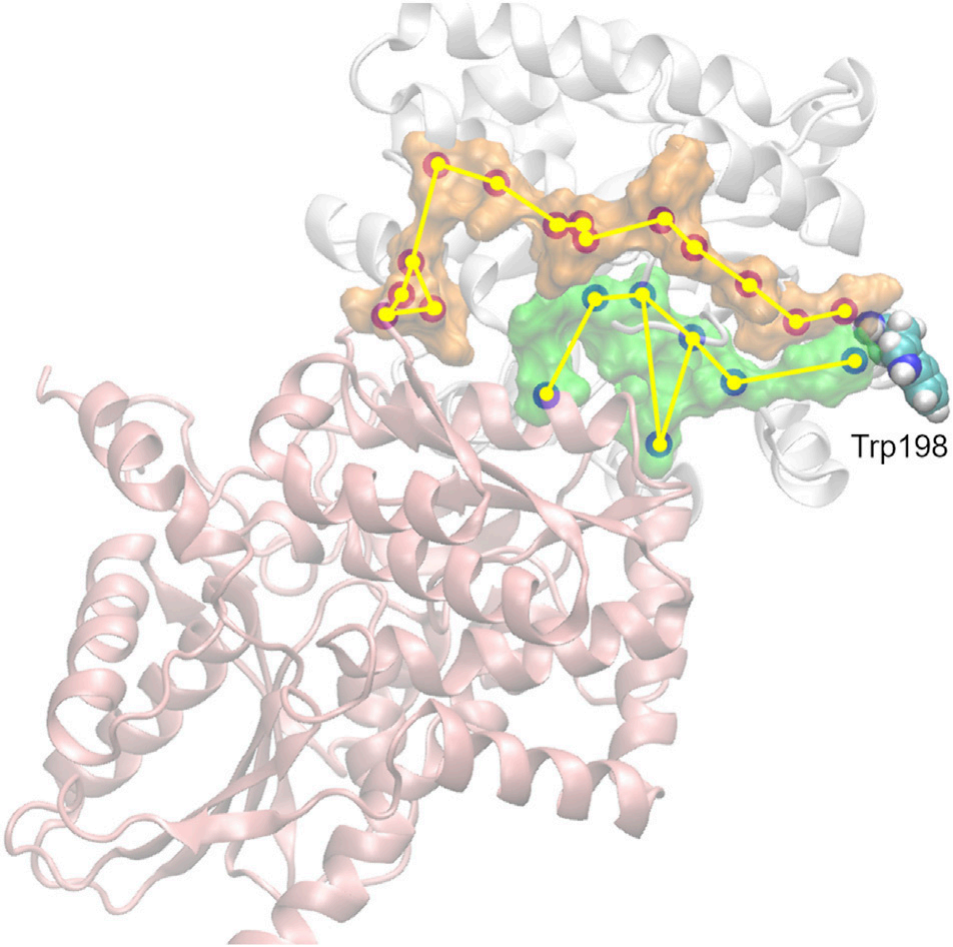
Propagation of interaction changes induced by the A198W substitution were examined in two directions leading to the αTS (white)—βTS (pink) interface. αC of residues with affected conformation are traced, based on pair-wise force distribution analysis, from the substitution site as follows: in orange–αAsn194, αLeu193, αLeu191, αAla189, αArg188, αGlu186, αAla185, αSer235, αLys239, αGlu242, αAsn66, αGln65, βSer161, βGly162; in green–αHis195, αPro156, βIle20, αLeu177, αPhe212, αThr183, βArg175.

**FIGURE 6:**
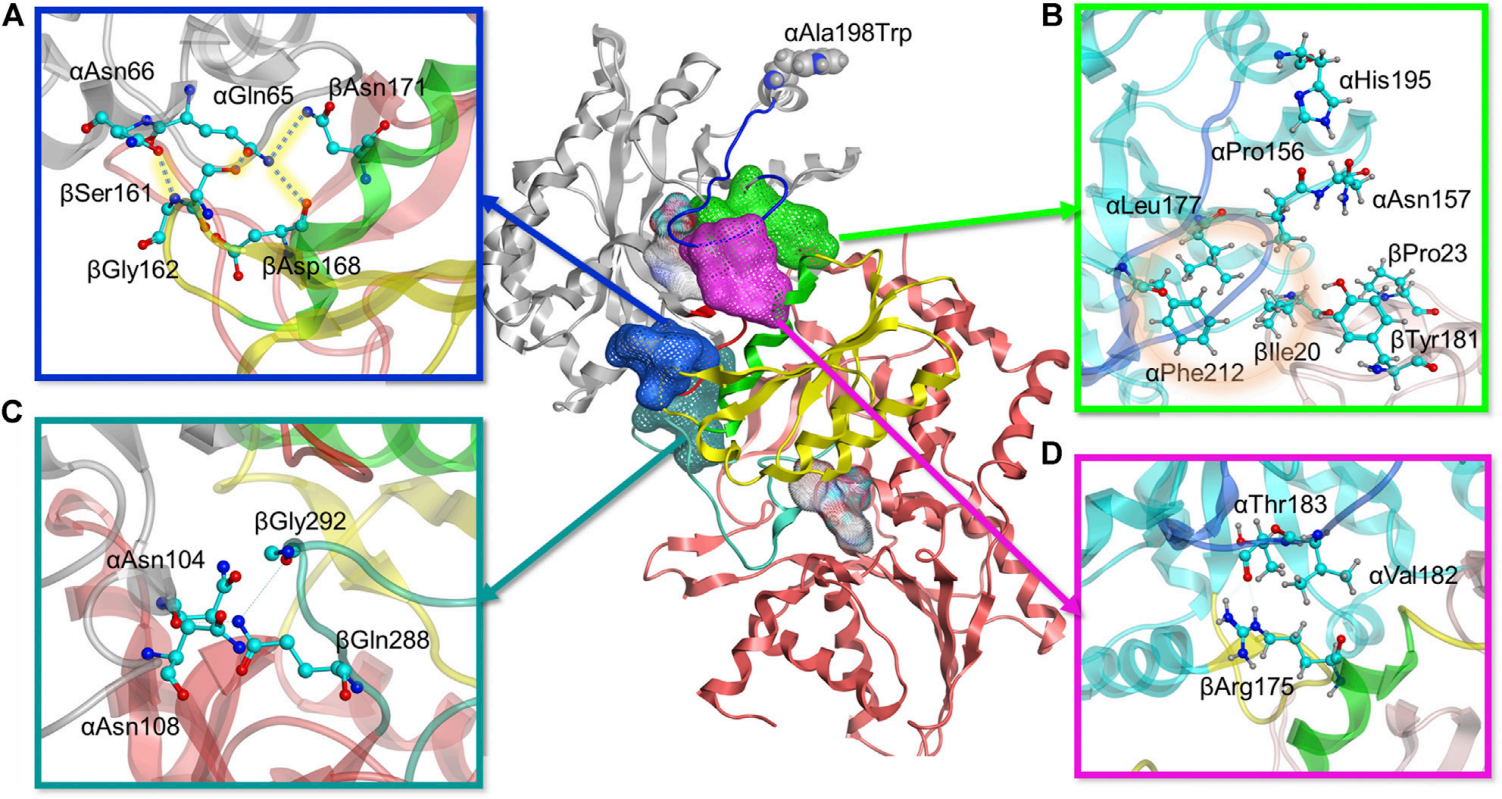
Molecular modeling reveals four positions with stronger interdomain interactions in A198W TS. **(A)** Electrostatic link between alpha residues αGln65, αAsn66 and beta residues βSer161, βGly162, βAsp168, and βAsn171 (also see Supplementary Figure 11). **(B)** Hydrophobic cluster between αLeu177, αPhe212, and βIle20 observed in A198W TS (also see Supplementary Figure 9). **(C)** αAsn104 forms consistent hydrogen bonds with βGln 288/βGly 292 in A198W TS **(D)** Consistent hydrogen bond between αThr183 and αArg175 may contribute to closed αL6 conformation (also see Supplementary 11A).

The MD simulations for the A198W variant were perhaps more insightful, and so, we focused more attention on analyzing these changes compared to what was observed for WT αTS. Briefly, the MD simulations indicated that local changes at the substitution site propagated structural dynamic changes throughout αTS (Figure 5), including decreasing the overall flexibility of αL6. It is it important to note that large scale conformational changes captured in our NMR experiments on millisecond timescale are induced by side chain motions and backbone fluctuations which could be detected within the nanoseconds timeframe. Detailed atomistic analysis of our trajectories explains how changes in forces and interactions lead to different dynamics and conformational behavior in the WT and A198W system. Our data shows that such changes led to the establishment of contacts at the αTS/βTS interface at four key positions in the A198W variant within the 200 ns simulation time, while such links were not formed or maintained in the WT enzyme on the same timescale (Figure 6). Furthermore, interactions observed for key residues within the indole channel (βTyr279, βPhe280) in the mutant system suggest that substrate channeling may be more efficient in the A198W variant. We detail some of the conformational dynamics and contact changes induced by the A198W substitution below.

As the simulations included both αTS and βTS subunits, we differentiate αTS and βTS residues and secondary structures by including the α and β designation in front of the secondary structure or residue. For example, the A198W substitution led to a displacement of α-helix 6 (αH6) towards αL6 (Supplementary Figure 8). This displacement allows αHis195 to form a dynamic but consistent hydrogen bond with αPro156 and a recurrent contact with αAsn157. A group of hydrophobic residues, αLeu177, αPhe212, and βIle20 were also in consistent close contact. While it is not clear if this set of interactions induces the helix shift or vice versa, lack of it, as observed in the WT system, resulted in a formation of a small cluster between αAsn157, βIle20, βPro23 and βTyr181. while the interactions between βIle20 and αLeu177 and αPhe212 were interrupted (Figure 6B, Supplementary Figure 9). This collection of interactions may be important as the absence of the hydrophobic contact between βIle20 and αLeu177 and αPhe212 may contribute to disordering of αL6, allowing it to move towards a more open conformation and providing a possible means of ligand escape from αTS (Supplementary Figure 9).

There were also additional changes to interactions involving αL6. For example, αAsn194 δN formed a more stable hydrogen bond with αAla226 O of helix 7 (αH7) in the A198W variant compared to WT TS. A backbone hydrogen bond between αLeu191 O and αGln210 N appeared to also be very strong in the A198W variant, while it was rarely observed in WT TS (Supplementary Figure 10). These findings were interesting considering that αGln210 and αAla226 appear to have suppressed conformational exchange on the millisecond timescale, at least according to the NMR studies on αTS alone (Figure 4). Consistent contact was also observed between αAla185, αSer215, and αSer235 which may favor the closed conformation of αL6 in the A198W variant, considering that these interactions were interrupted in the WT enzyme.

A very critical aspect of the dynamics of αL6 in the A198W system was its consistent interactions with helix 6 of βTS (βH6), an important part of the COMM domain, well known to play a key role in the interdomain communication and activation of αTS (Dunn, 2012). Pairwise force data and hydrogen bond analysis showed a consistent hydrogen bond between αThr183 O and βArg175 guanidino group in over 70% оf the frames analyzed. This hydrogen bond was not maintained in any of the trajectories of the simulated WT system (Figure 6D, Supplementary Figure 11). In addition to this hydrogen bond, a hydrophobic interaction was established between αVal182 and the hydrocarbon portion of the βArg175 sidechain. Loss of these interactions as observed in the WT system and in one of the trajectories for the A198W system resulted in displacement and disordering of αL6. In the A198W trajectory with the absent hydrogen bond between αThr183 and βArg175, a hydrophobic interaction between αVal182 and βSer178 and βGly179 was maintained but it was not sufficient to prevent the loop displacement.

Another series of interactions correlated with the events occurring at the αL6 and βH6 interface - interactions of αGln65 and αAsn66 with βSer161 and βGly162 (Figure 6A). Dihedral analysis showed one distinct conformation for the C – αC – βC – γC angle of αGln65 and αAsn66 in the A198W variant (Supplementary Figure 11). This conformation was associated with a hydrogen bond between αGln65 and βSer161 (68% in A198W and less than 3% in WT). In addition, electrostatic (salt-bridge like) interaction between αGln65 and beta residues βAsp168 and βAsn171 was observed in the A198W variant. βAsp168 and βAsn171 were also part of βH6. A hydrogen bond between δO of αAsn66 and the backbone of βGly162 was observed in over 50% of all frames for the A198W system, and less than 20% in the WT enzyme. Another hydrogen bond between the backbone nitrogen of αGln65 and αGly61 was also more stable in the A198W variant. These results are interesting considering that the A198W substitution induced substantial chemical shift changes in both αGly61 and αAla67 (Figure 3). Unfortunately, the backbone resonances of αGln65 and αAsn66 are unassigned. Nonetheless, the NMR results indicate that there are substantial structural dynamic changes in this region even in the absence of βTS. The A198W substitution also induced millisecond conformational exchange in αAla67 (Figure 4), suggesting that this region may be seeking alternative binding interactions.

**FIGURE 7:**
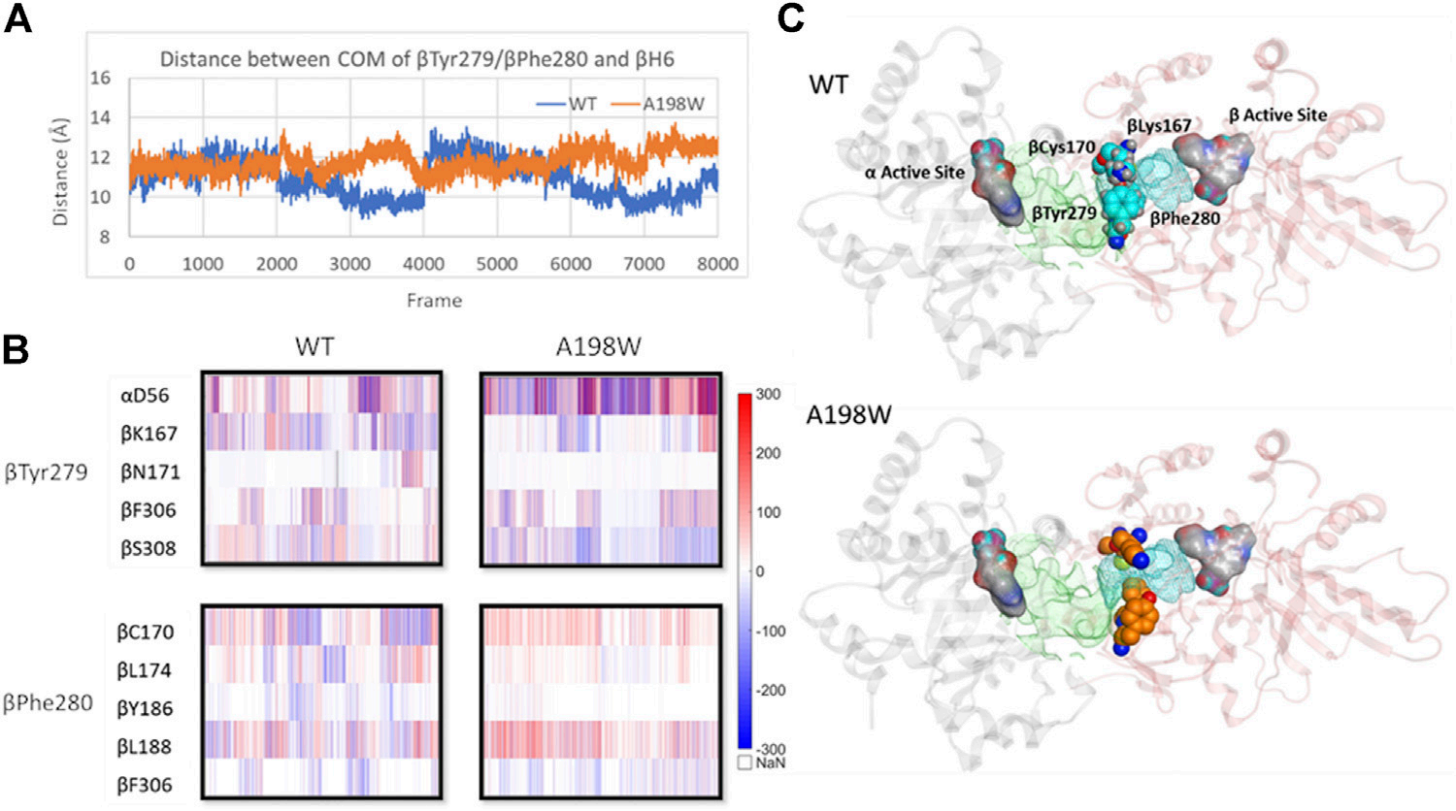
Displacement of βH6 towards αTS leads to widening of indole channel which is further supported by weaker interactions between the channel lining residues βTyr279 and βPhe280 with residues of βH6 in A198W. **(A)** Distance between center of mass of residues βTyr279/βPhe280 and βH6 lining the affected tunnel portion **(B)** Pairwise force distribution analysis indicates that interactions for residues βTyr279 and βPhe280 have different trends in WT and A198W. **(C)** Interactions between βTyr279 and βLys167 position the aromatic ring within the indole channel. Active sites in both subunits (grey–α subunit, pink–β subunit) are represented as atom colored surface. Indole path is shown in green surface and the affected portion of the channel is mapped in cyan. See also Supplementary Figure 12.

The interactions of αGln65 and αAsn66 with the small loop between beta sheet 5 (βS5) and beta helix 6 (βH6) and αThr183-βArg175 hydrogen bond appeared to also affect the position of βH6 relative to αTS. In the A198W variant the displacement occurred in the direction of αTS whereas in WT enzyme, lack of consistent interactions led to displacement in the opposite direction away from the αTS (Supplementary Figure 12). The difference in the βH6 position also affected important residues lining the indole channel, namely βTyr279 and βPhe280. Force analysis showed that both residues in the WT enzyme have stronger interactions with residues from βH6. A more consistent link between βTyr279 and βLys167 was observed in WT TS. A hydrogen bond between βTyr279 and βAsn171 was also observed in one of the WT simulations. Such interactions position the phenol ring of βTyr279 within the tunnel possibly interfering with the indole transfer (Weyand and Schlichting, 2000). Similarly, βPhe280 in WT enzyme showed stronger interactions with βCys170 of βH6 which brought the phenyl sidechain within the indole channel. In the A198W variant, these residues favored interactions with beta residues 306–308 on the channel “wall.” A hydrogen bond between βTyr279 and αAsp56 was observed in 40% of MD frames in the A198W variant compared to 10% in WT TS. Such interaction may stabilize the phenol ring of βTyr279 in an “open channel” conformation (Figure 7).

Other interactions induced by the A198W substitution may also be important for αTS-βTS communication, although the mechanism is unclear. For example, αAsn104 favors interactions with βTS in the A198W variant (Figure 6C). In WT TS, αAsn104 formed a very consistent hydrogen bond with αAsp130 ( > 60% frames analyzed) and in much smaller extent with the backbone of βIle278. In the A198W variant, a dynamic hydrogen bond network formed between αAsn104 and βGln 288/βGly 292; these beta residues form part of the allosteric “metal binding loop” (Weyand and Schlichting, 1999). NMR studies indicated that the A198W substitution induced chemical shift perturbations for αAsn104 and αAsp130.

## Discussion

Amino acid interactions networks have been proposed to be the means through which allosteric signals are propagated across a protein’s structure (Dokholyan, 2016). Understanding such networks has practical importance in protein engineering (Lee et al., 2008; Reynolds, McLaughlin and Ranganathan, 2011; Gorman et al., 2019) and allosteric drug design (Nussinov and Chung, 2015; Greener and Sternberg, 2018). It should be noted that Hilser and colleagues have proposed allosteric models that do not require “networks” to transmit information across a protein framework. Instead, these models focus on changes within the conformational ensemble using a free energy landscape framework (Motlagh et al., 2014). However, we believe these views of allostery can be compatible (D’Amico et al., 2020). In fact, the CHESCA approach identifies network residues as those being involved in the same conformational change (Selvaratnam et al., 2011), but does not necessitate that there are direct interactions between such residues.

We have previously identified networks in αTS using a CHESCA-type approach (Axe and Boehr, 2013; Axe et al., 2014; O’Rourke et al., 2019), and have found that network substitutions can have detrimental effects on αTS catalytic activity (Axe et al., 2014). We developed a cell-based assay to screen additional αTS variants by connecting function to cellular growth rates. We found that the surface-exposed, residue position 198, distant from both the αTS active site and the αTS/βTS interface (Figure 1), was sensitive to amino acid substitutions. Specifically, we discovered αTS variants (A198K, A198V) that failed to support growth of tryptophan auxotrophic *E. coli* cells and discovered a variant (A198W) that surprisingly enhanced the growth rate of these cells (Figure 2). While the enhanced function was modest, it was similar to the enhancement of βTS activity by previous protein engineering efforts by the Arnold lab (Buller et al., 2015; Murciano-Calles et al., 2016). The A198W substitution likely enhances TS activity by modulating conformational dynamics involving other network residues (Figures 3, 4) to communicate with βTS (Figures 5, 6) and help open the indole channel (Figure 7). It is remarkable that the effect of the A198W substitution is only realized in the full TS complex, suggesting that the intrinsic networks we previously identified in αTS alone have functional roles in the full TS enzyme. It will be likewise informative to evaluate networks in βTS using the same methods we have developed for αTS, as well as further test the αTS network using the methods developed here.

We note that the rate limiting step for the αTS reaction seems to be a conformational change that occurs after IGP binds, and this step is sped up significantly in the presence of serine-bound βTS (Anderson et al., 1991). For the βTS reaction, the rate limiting step is the proton abstraction from the external aldimine, which is also highly influenced by the presence of ligand-bound αTS (Ngo, et al., 2007). For the complete TS reaction, it is postulated that the release of tryptophan is at least partially rate limiting. In this context, and given the kinetic, NMR and MD simulation results, it would seem that the A198W substitution enhances TS function by modulating dynamic interactions at the αTS-βTS interface, which promotes βTS activity, possibly enhancing release of tryptophan product. It is also enticing to suggest that the substitution boosts indole channeling, due to the higher enhancements of the full TS reaction compared to the βTS reaction alone, and the indication in the MD simulations that the indole channel is more open, more often in the A198W variant. In a similar manner, it has been suggested that the enhancing mutations in *P. furiosus* βTS allosterically modulate conformational dynamics related to the conformational cycle necessary for the complex βTS reaction (Maria-Solano et al., 2019). We also note that most of the amino acids involved in the αTS-βTS interaction and/or form the indole channel are conserved (see Supplementary Figures 13 and 14) or have co-evolved to maintain interactions (see Supplementary Figure 6). While Ala198 itself is not conserved, other residues in the NMR-derived network are conserved or co-evolve (see Supplementary Figure 6), and so similar networks might exist in these other TS enzymes. Such conformational dynamics and allosteric networks may be important for the evolution of the communication between the αTS and βTS subunits (Schupfner et al., 2020). As such, other network substitutions in *E. coli* αTS and other αTS enzymes might likewise activate TS function through a similar mechanism.

More generally, these results indicate that amino acid interaction networks may not only be important for function within an enzyme subunit, but they may be used to bridge communication between subunits in a multienzyme complex, and only reveal such enhancements in the full complex. This finding reveals new possibilities for controlling function in such complexes; both individual enzyme function and communication between subunits might be modulated through engineering network residues. Similar studies have been performed on imidazole glycerol phosphate synthase (Rivalta et al., 2012; Lisi et al., 2016; Lisi et al., 2017), which is a histidine biosynthetic, bifunctional enzyme also demonstrating substrate channeling. Our results reported here are unique in that the network substitution enhanced enzyme function, atypical for any mutational study, and the substitution was surface-exposed. Modifying surface-exposed network residues are unlikely to change the protein fold or substantially affect protein stability, and thus, offer a novel and potentially useful strategy in enzyme design for overall rate enhancement.

Networks in αTS not only connect the αTS active site to the αTS/βTS interface, but also connect to other protein surfaces (Axe et al., 2014; O’Rourke et al., 2019). While these network surfaces might be targeted by novel allosteric modulators (Nussinov and Chung, 2015; Greener and Sternberg, 2018) or be used to graft novel regulatory units (Lee et al., 2008; Reynolds et al., 2011; Dokholyan, 2016), it is also intriguing to ask why these network surfaces even exist. Are allosteric networks simply intrinsic to all proteins (Gunasekaran et al., 2004)? Or has evolution shaped these allosteric surfaces for other purposes? For example, metabolites or other enzymes involved in tryptophan metabolism might interact, albeit weakly, with these network surfaces to modulate TS function. The fast cell growth phenotype engendered by the A198W substitution might partially or fully arise because it modifies unidentified protein-protein interactions, instead of owing solely to changes to enzyme activity. Other “quinary” interactions (Cohen and Pielak, 2017) may likewise exert their effects through network surfaces on other proteins. NMR, MD, high throughput mutational screening (Wrenbeck et al., 2017) and other network methods (O’Rourke et al., 2016) can be used to evaluate the physical basis and molecular evolution of networks in other enzyme complexes, potentially revealing how allosteric networks connect the larger proteome (Cong et al., 2019).

## Data Availability

The original contributions presented in the study are included in the article/Supplementary Material, further inquiries can be directed to the corresponding author.
