# Supplementary material for: Substitution of a Surface-Exposed Residue Involved in an Allosteric Network Enhances Tryptophan Synthase Function in Cells
Source: Front Mol Biosci. 2021 May 26;8:679915. doi: 10.3389/fmolb.2021.679915 (PMC8187860; doi:10.3389/fmolb.2021.679915)
Supplement: Supplementary file 1 [file DataSheet1.PDF]

Supplementary Material

for

Substitution of a surface-exposed residue involved in an allosteric network enhances tryptophan synthase function in cells

Rebecca N. D'Amico[1,†], Yuliana K. Bosken[2,†], Kathleen F. O'Rourke[1], Alec M. Murray[1], Woudasie Admasu[1], Chia-en A. Chang[2] and David D. Boehr[1*]

[1]Department of Chemistry, The Pennsylvania State University, University Park, PA, USA

[2]Department of Chemistry, The University of California Riverside, Riverside, CA, USA

[†]these authors have contributed equally to this work and share first authorship

Supplementary Table 1. Kinetic and thermodynamics parameters for the millisecond motions in WT and A198 αTS at 283 K

Protein	State	k_{ex} (s^{-1})	p_A (%)	p_B (%)	$k_{A\rightarrow B}$ (s^{-1})	$k_{B\rightarrow A}$ (s^{-1})
WT[1]	*resting*	286 ± 58	94.2	5.8	17	269
	working	273 ± 19	94.4	5.6	15	258
A198W	*resting*	156 ± 18	93.2	6.8	11	145
	working	185 ± 15	92.3	7.7	14	171

[1] from O'Rourke *et al*, 2018. Frontiers in Molecular Biosciences, 5, 92.

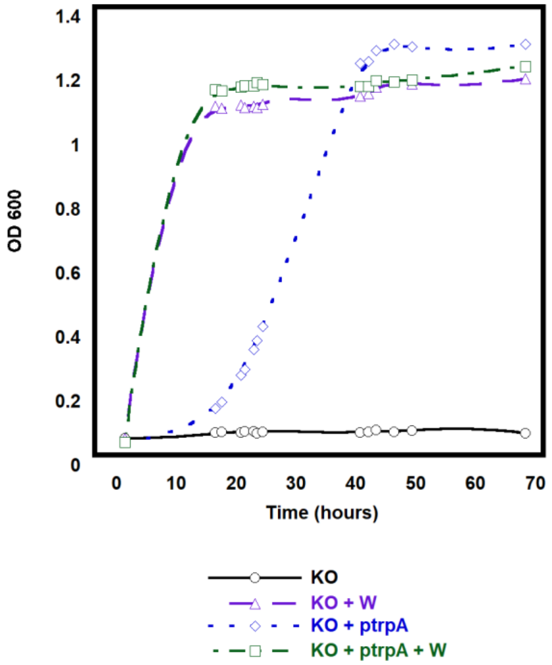

Supplementary Figure 1. Establishing parameters for the cell-based assay of tryptophan production. E. coli K12 cells with the trpA gene knocked out (KO) were incubated with and without tryptophan and with and without a plasmid encoding the wild type trpA enzyme. Cells with no plasmid or tryptophan supplements failed to grow (black); cells supplemented with tryptophan with and without the plasmid grew equivalently (green and blue, respectively); cells supplemented with plasmid and no tryptophan began doubling at a later time than the cells supplemented with tryptophan, but ultimately reached similar final OD values (blue).

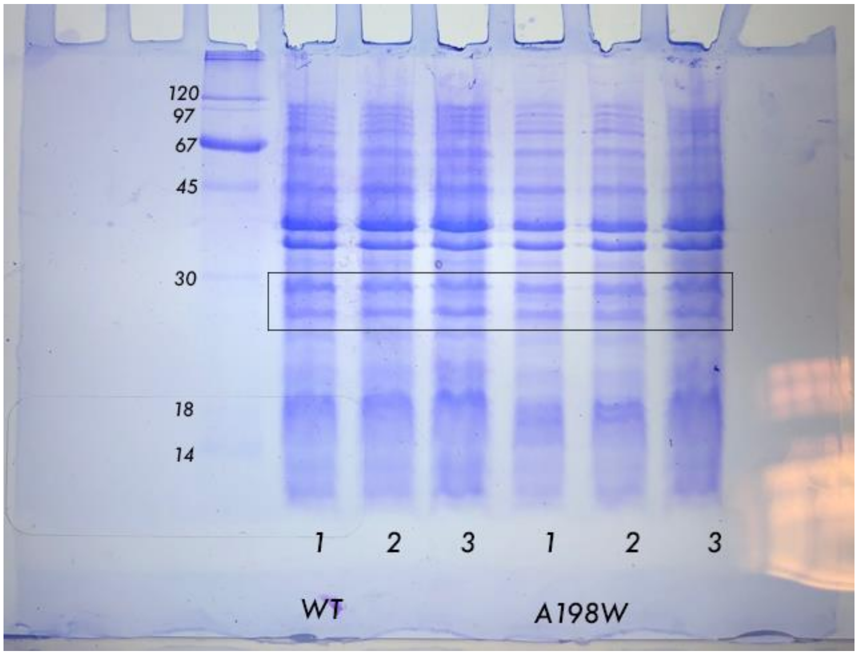

Supplementary Figure 2. SDS-PAGE analysis of αTS contained within the cells complemented with the WT and the A198W αTS expressing plasmid**.** No significant difference was visually detected for expression of WT and A198W αTS. Shown are three independent trials for both WT and A198W αTS expressing cells.

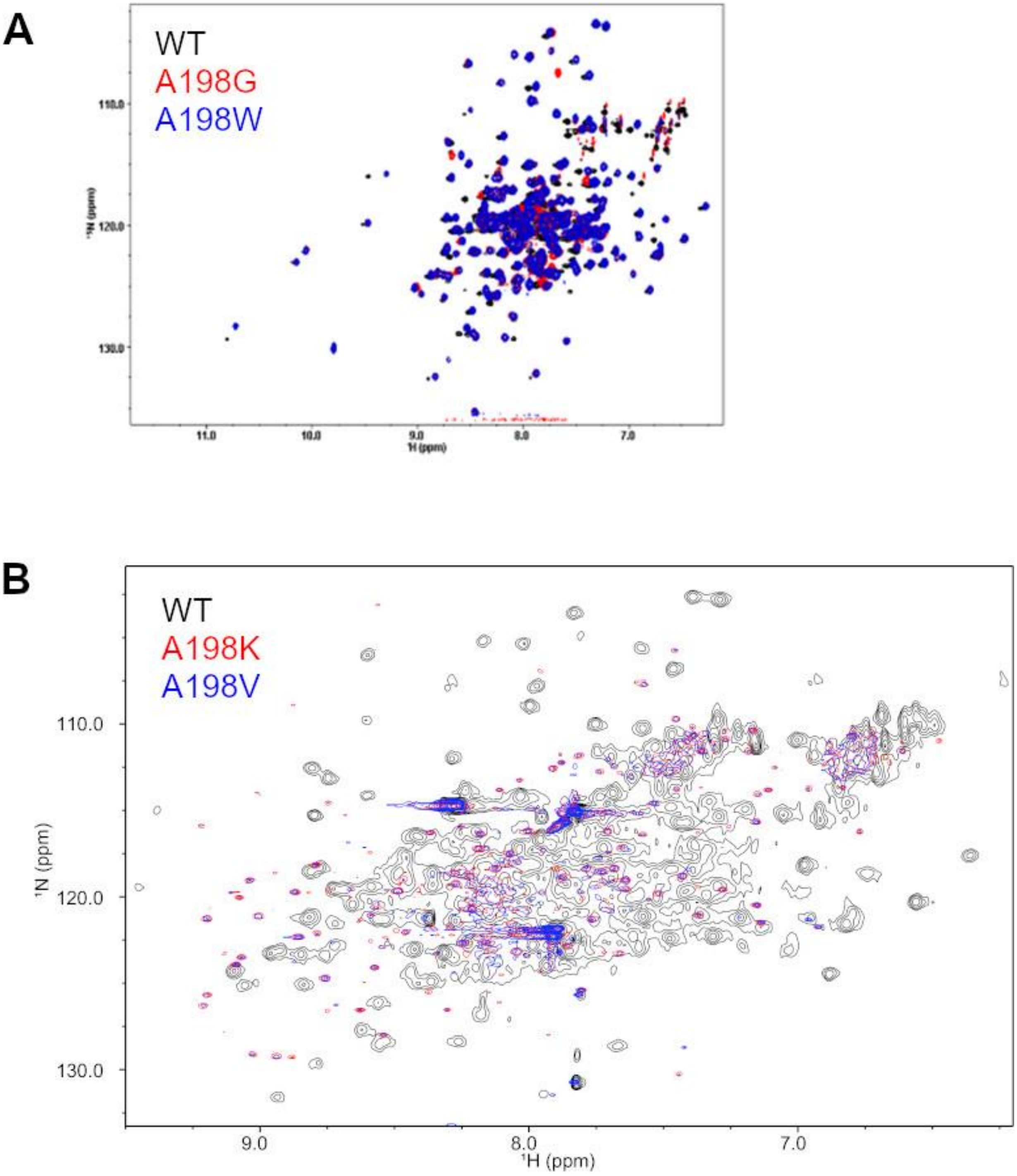

Supplementary Figure 3. NMR ^{1}H-^{15}N HSQC comparisons between WT and A198 variants. (**A**) NMR spectra comparisons between WT (black), A198G (red) and A198W (blue) αTS. Complementation of trpA-deleted *E.coli* cells with plasmid expressing A198G and A198W substitutions lead to enhanced growth, although only the results for A198W are statistically significant. (**B**) NMR spectra comparisons between WT (black), A198K (red) and A198W (blue) αTS. Plasmids expressing the A198K and A198V variants fail to rescue *E.coli* growth in the absence of tryptophan.

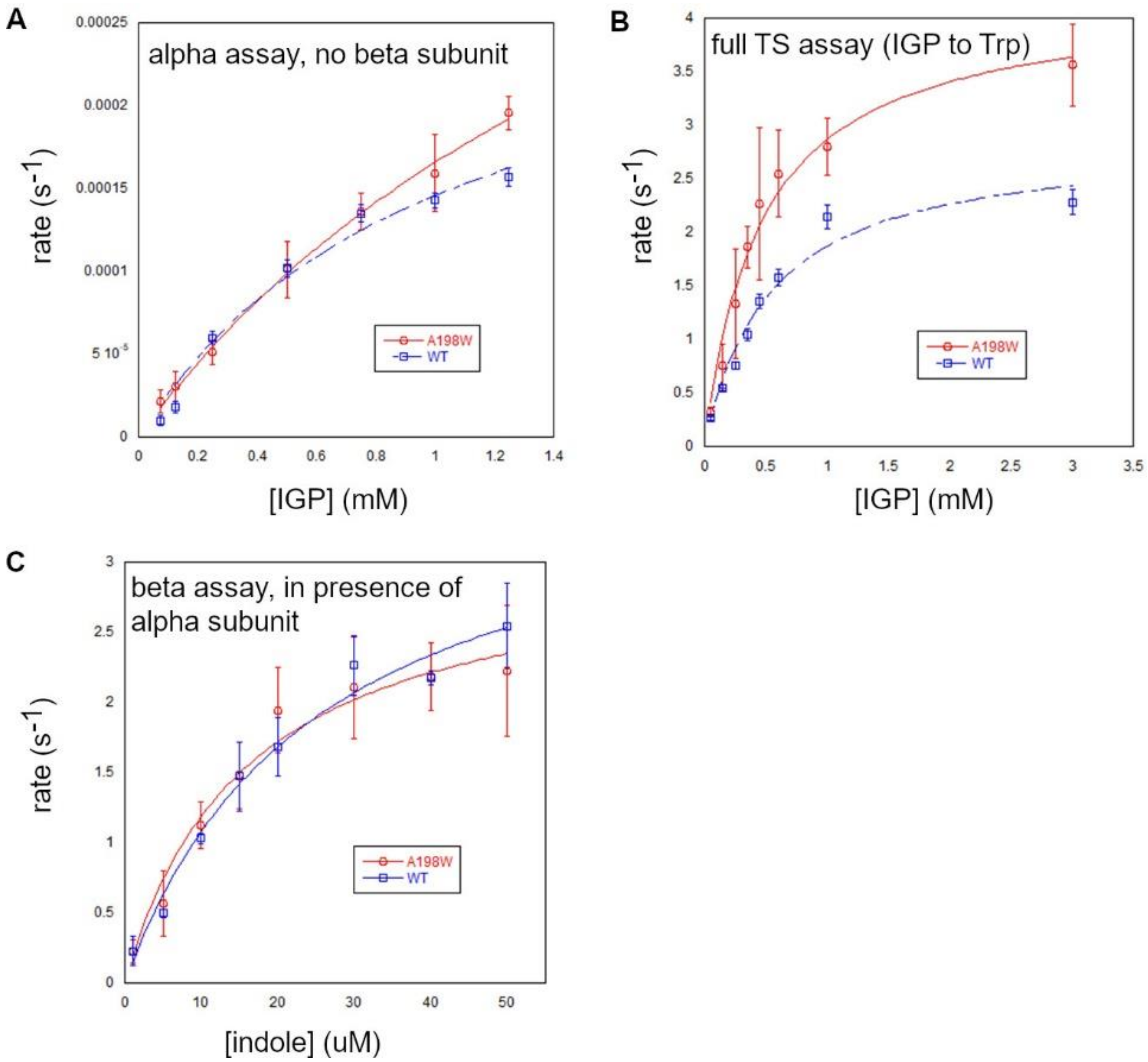

Supplementary Figure 4. Representative initial reaction rates versus substrate concentrations for WT (blue) and A198W (red) assays. (**A**) The alpha subunit reaction was assayed in the absence of the beta subunit. (**B**) The full TS reaction was assayed, starting from IGP and ending with tryptophan product. (**C**) The beta subunit reaction (initiated with indole and ending with tryptophan product) was assayed. Each data point was performed in at least triplicate.

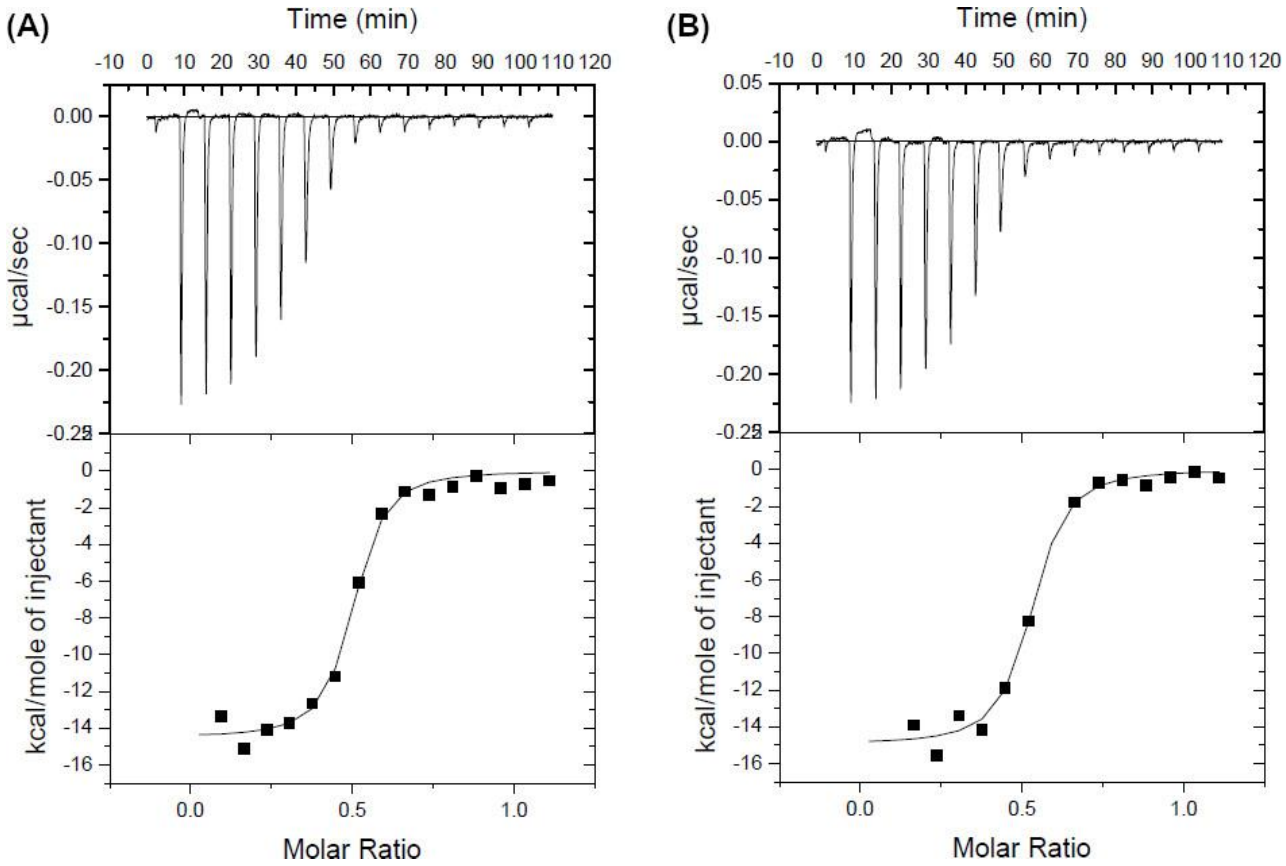

Supplementary Figure 5. The A198W substitution does not change binding affinity between αTS and βTS. Isothermal titration calorimetry experiments with (**A**) WT (K_d = 25±6 nM; ΔH = -14.52±0.33 kcal mol^{-1}; N=0.475±.007) and (**B**) A198W (K_d = 28±8 nM; ΔH = -14.96±0.38 kcal mol^{-1}; N=0.503±.009). A binding ratio (n) of 0.5 is expected as βTS was treated as a dimer for the αββα complex.

A

Residue	Frustration Index			
	PDB 1K3U	PDB 1C9D	PDB 5CGQ	PDB 3CEP
Glu2	-2.227	-1.950	-1.927	-1.941
Pro28	-1.342	-1.259	-1.342	-1.338
Gln32	-1.297	-1.295	-1.291	-1.303
Asp46	-1.366	-1.388	-1.435	-1.400
Asp56	-1.381	-1.230	-1.380	-1.570
Asn66	-1.258	-1.315	-1.363	-1.326
Glu83	-1.239	-1.237	-1.240	-1.211
Asp130	-1.517	-1.462	-1.490	-1.517
Glu135	-1.322	1.282	-1.259	-1.261
Lys263	-1.708	-1.384	-1.759	-1.211

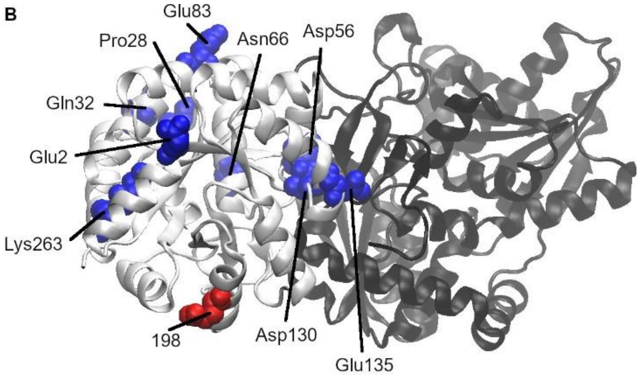

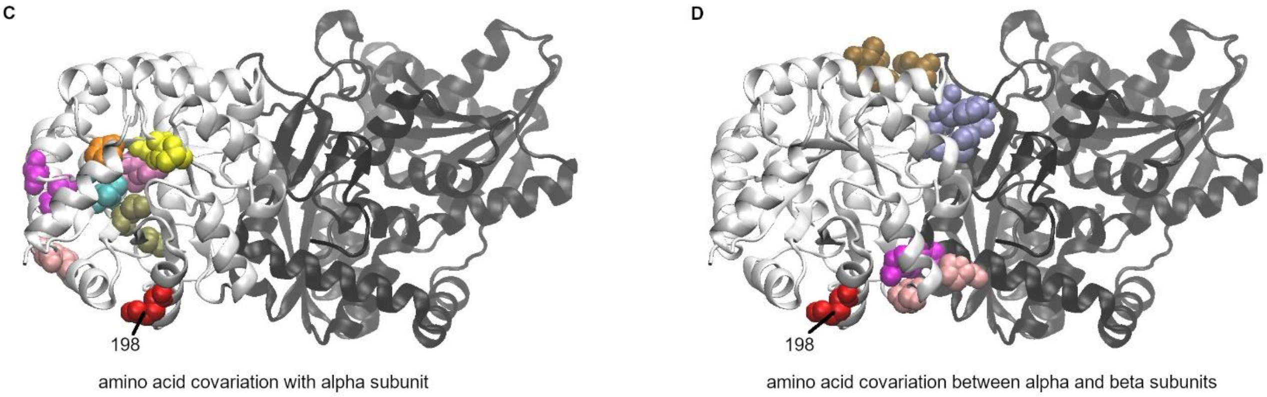

Supplementary Figure 6. Bioinformatic analysis of the NMR-derived networks. (**A**) Single-residue level frustration indices determined according to the AWSEM-MD Frustratometer (http://frustratometer.qb.fcen.uba.ar) using different PDB structures of the *S. typhimurium* TS as input values. Single residues are determined as highly frustrated if the frustration index is less than -1, which would indicate that most other amino acids at that location would be more favorable for folding than the native ones by more than one standard deviation of that distribution. Most of the highly frustrated residues are similar among the different TS structures, including the ones listed here. (**B**) Highly frustrated residues mapped onto the *S. typhimurium* TS structure (PDB 1K3U). (**C and D**) Pairs of covarying/coevolving residues according to the RaptorX server (http://raptorx.uchicago.edu/ComplexContact) using *E.coli* αTS and βTS as input sequences. Amino acid covariation pairs within αTS (see panel C) include (*E.coli* identity and numbering): Ala43-Val259 (magenta), Ala47-Pro96 (orange), Ser125-Ala149 (yellow), Gly98-Ser125 (yellow), Gly211-Ile232 (tan), Gly51-Leu100 (green), Pro217-Ala265 (pink), Val20-Ala47 (cyan) and Phe22-Glu49 (mauve). Amino acid covariation pairs between αTS and βTS (see panel D) include (*E.coli* identity and numbering): αTS Thr77, Pro78 and Ala79 covary with βTS Asp291 (ochre), αTS Ala103, Asn104 and Phe107 covary with βTS Gly277 and Ile278 (blue), αTS Pro155 covaries with βTS Ile20 (magenta), and αTS Asn157 covaries with βTS Pro23 (pink). It is notable that all paired residues make contact with their partners.

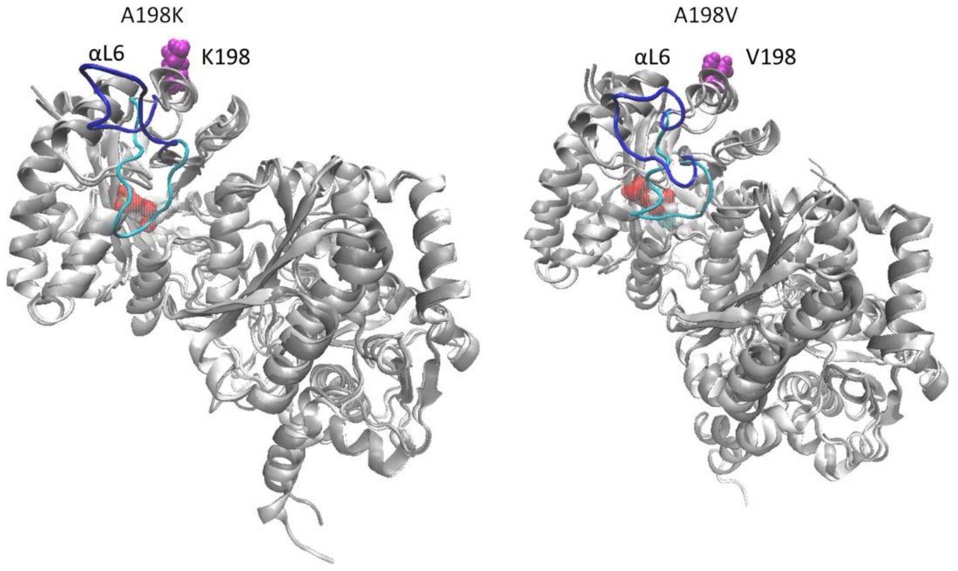

Supplementary Figure 7. Molecular dynamics simulations suggest that the A198K and A198V induce a large conformational change in the active site αL6 (light and dark blue). Shown are snapshots from the MD simulations for the A198K (left) and A198V (right) variants.

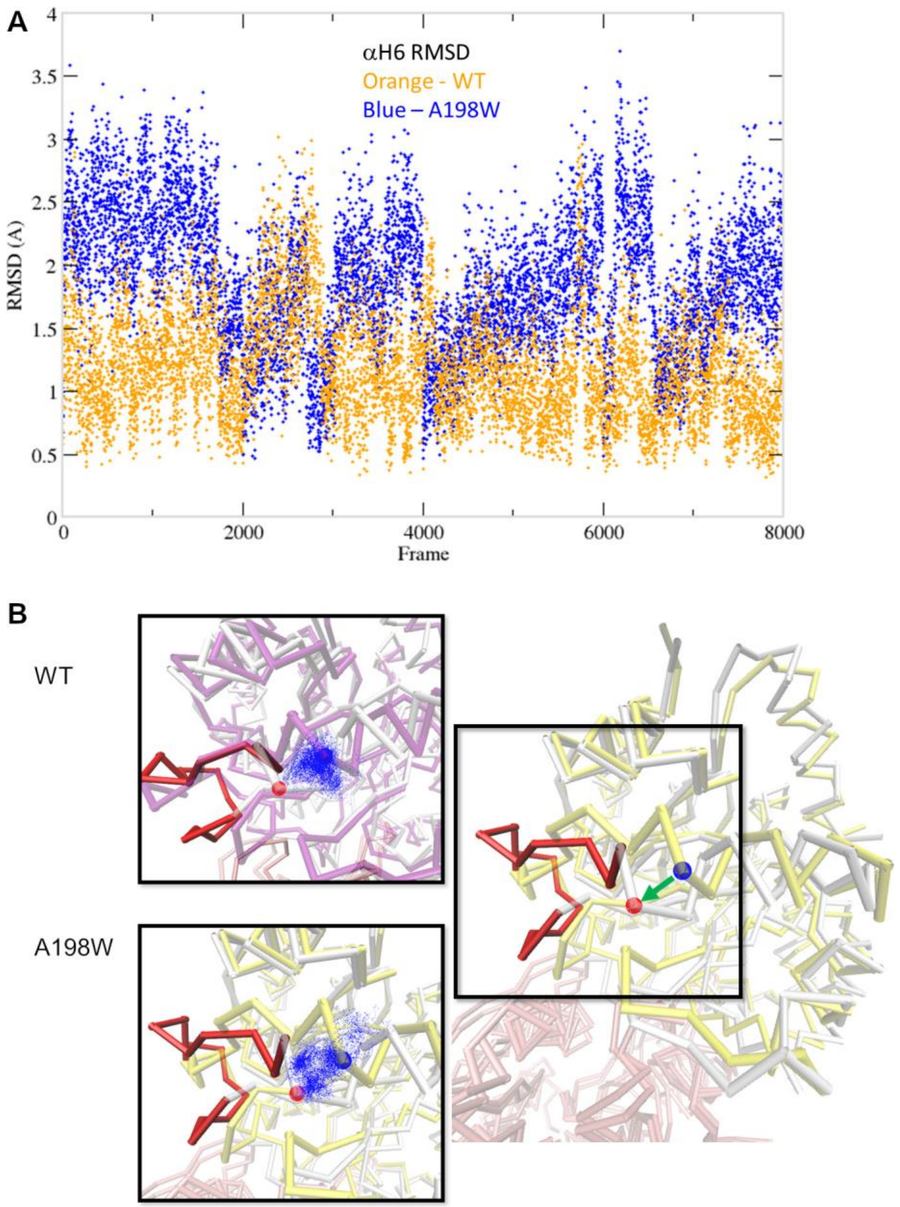

Supplementary Figure 8. Displacement pattern of αH6 leads to conformation change in local and distant residues. (**A**) Root-mean-square deviation (RMSD) for αC of αH6 (residues 194 – 203). (**B**) αHis195 αC position distribution over 8000 analyzed frames represented by blue dots. The initial position of the atom is shown in blue and the most distant position in red. αL6 is colored in red.

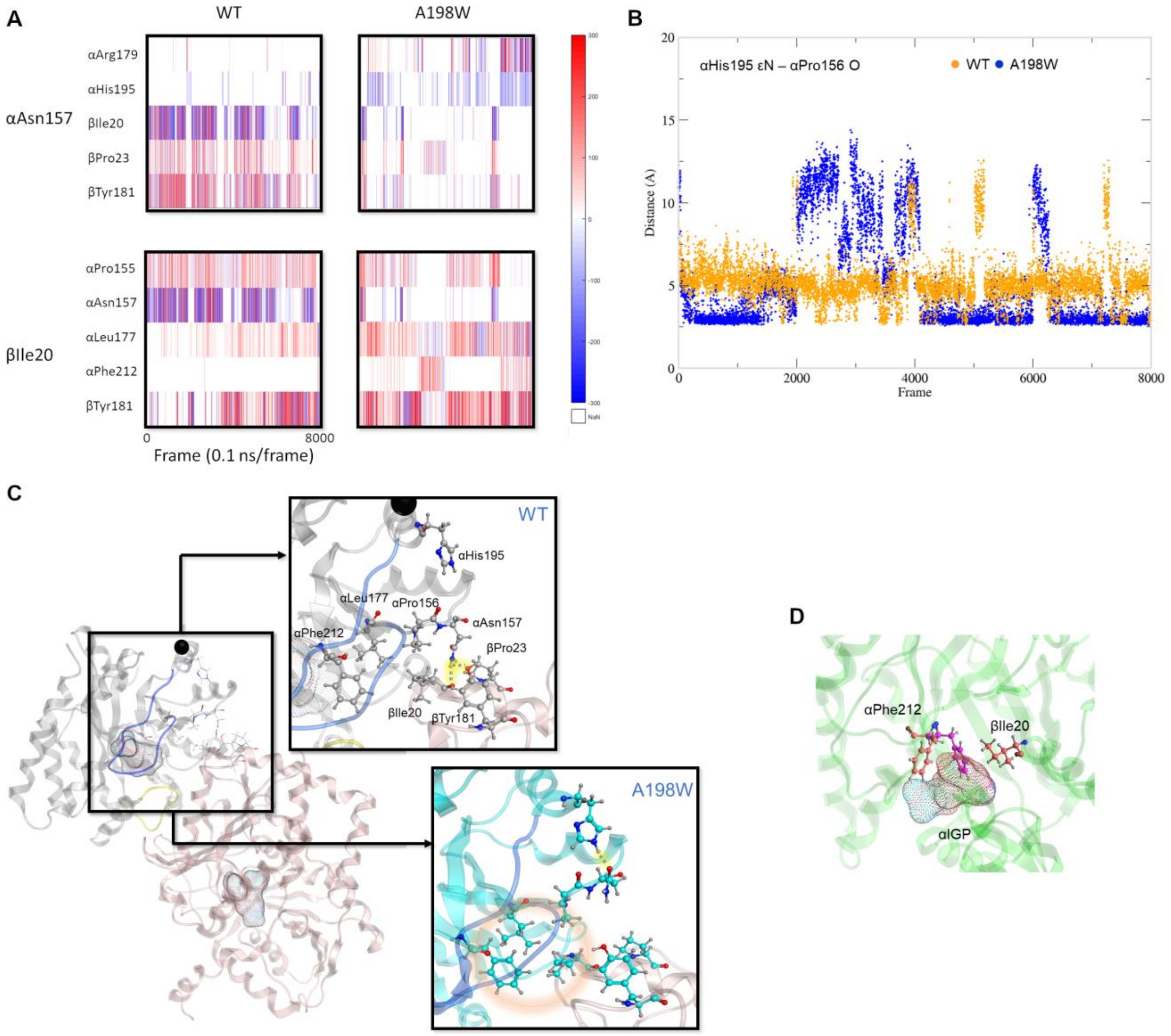

Supplementary Figure 9. Local interaction changes are induced by A198W substitution. Pair-wise force distribution analysis (**A**) shows different interactions for αAsn157 and βIle20 in WT and A198W TS. Hydrogen bond between αHis195 and αPro156 (**B**) is formed and maintained in three of the A198W MD simulations analyzed, while this hydrogen bond is rarely observed in WT. Hydrophobic interactions between residues βIle20 (βTS in pink), αLeu177 and αPhe212 in A198W are inconsistent in WT (**C**). Lack of such interactions may lead to rotation of the sidechain of αPhe212 providing escape route for substrate (**D**).

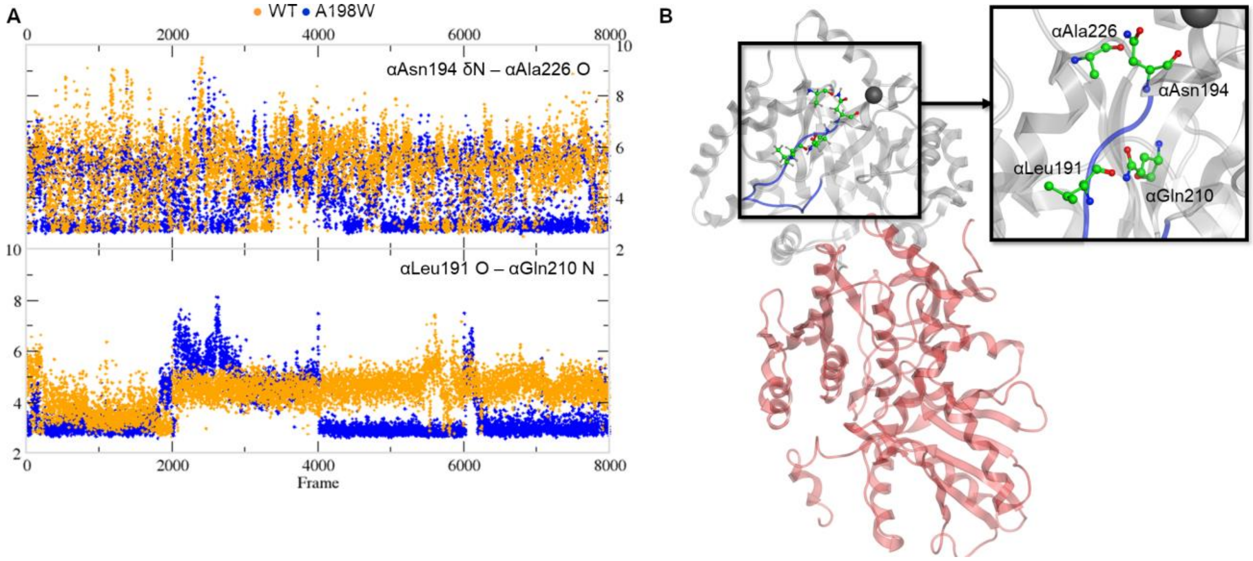

Supplementary Figure 10. Hydrogen bonds between αAsn194 and αAla226, and αLeu191 and αGln210 (**A** and **B**) are more consistent in A198W and may further contribute to the ordered closed conformation of αL6 (**B**) colored in blue. Substitution site is indicated by black circle, αTS is colored in white and βTS is colored in pink.

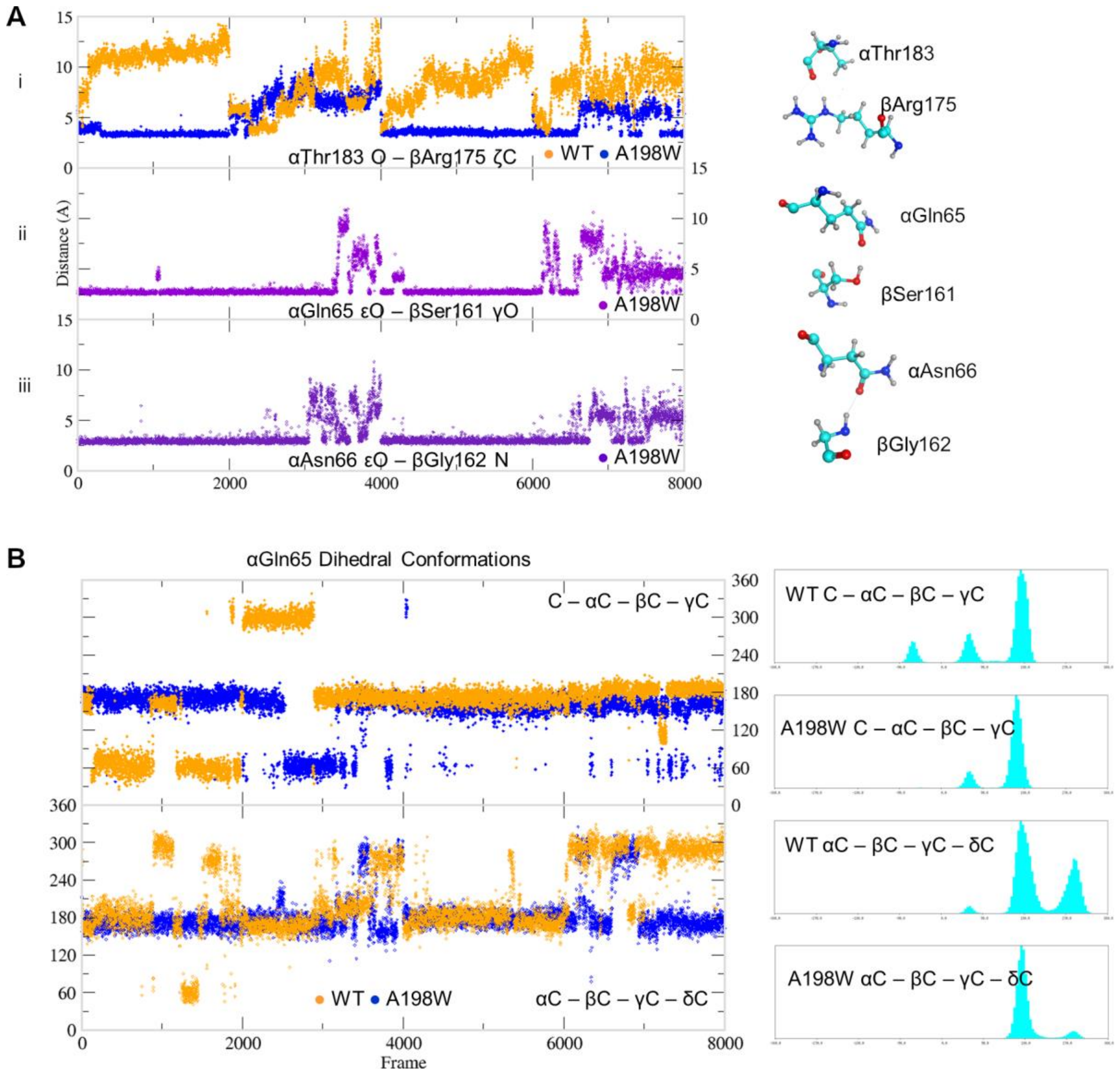

Supplementary Figure 11. Hydrogen bonds at αTS/ βTS interface in A198W (**A**) are more consistent and affect the conformation populations of the involved residues. Dihedral analysis shows one predominant conformation for αGln65 in A198W and multiple conformations in WT due to lack of stable interactions (**B**).

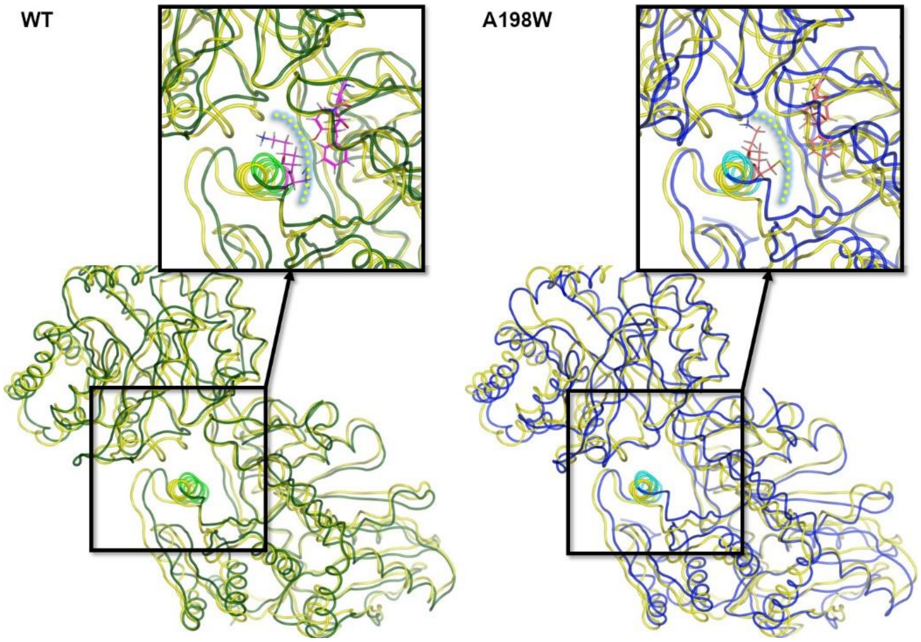

Supplementary Figure 12. Displacement of βH6 towards αTS leads to widening of indole channel which is further supported by weaker interactions between the channel lining residues βTyr279 and βPhe280 with residues of βH6 in A198W. Initial frame of MD simulations for both systems is in yellow trace and consequent displacement indicated in green for WT and blue for A198W, βH6 is colored in light green and light blue, respectively.

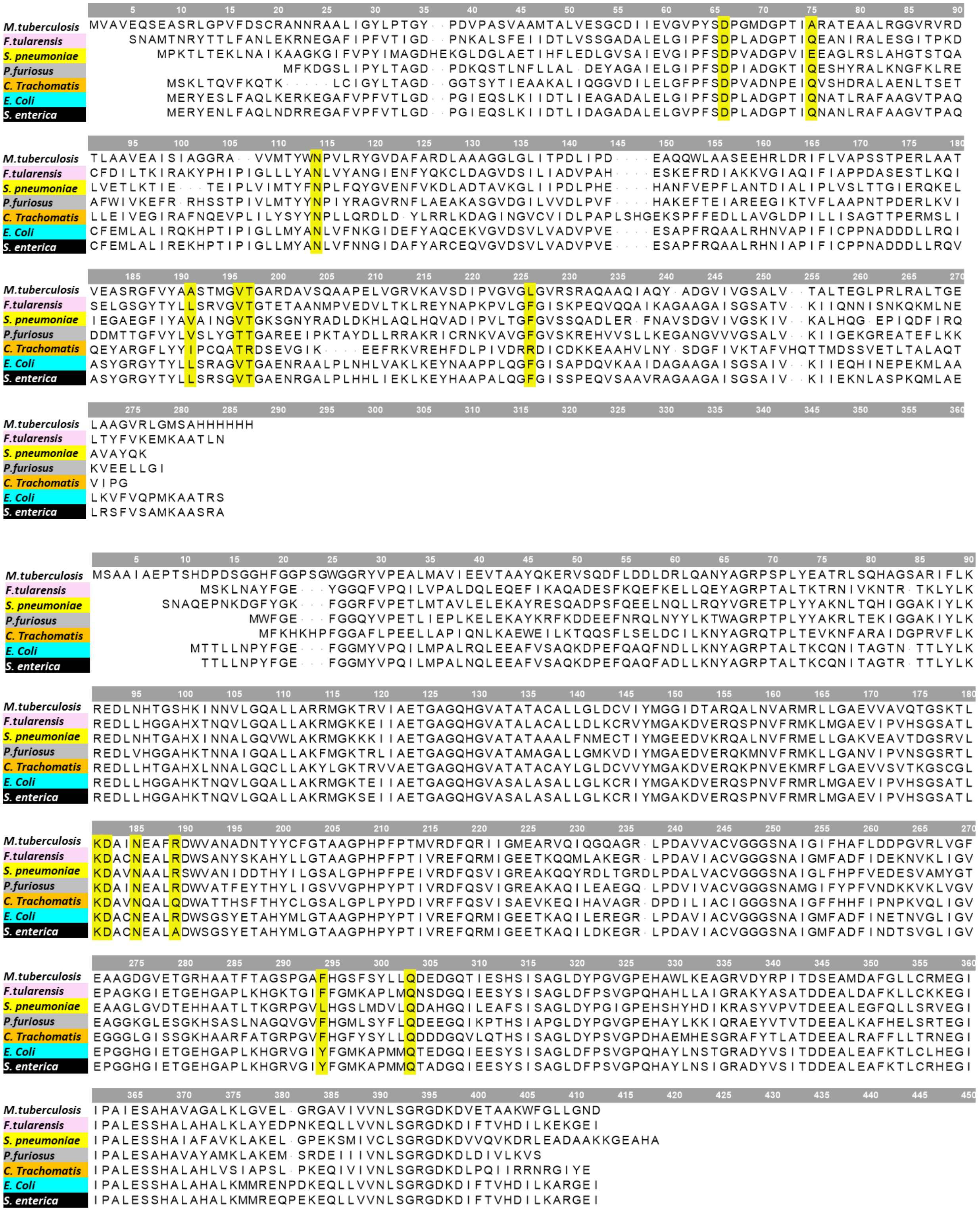

Supplementary Figure 13. Sequence alignment for (**A**) α and (**B**) β chains of TS from *M. tuberculosis* (white), *F. tularensis* (pink), *S. pneumoniae* (yellow), *P. furiosus* (grey), *C. trachomatis* (orange), *E. coli* (teal), and *S. enterica* (black). Conserved residues, showing conformational changes due to the A198W substitution are highlighted.

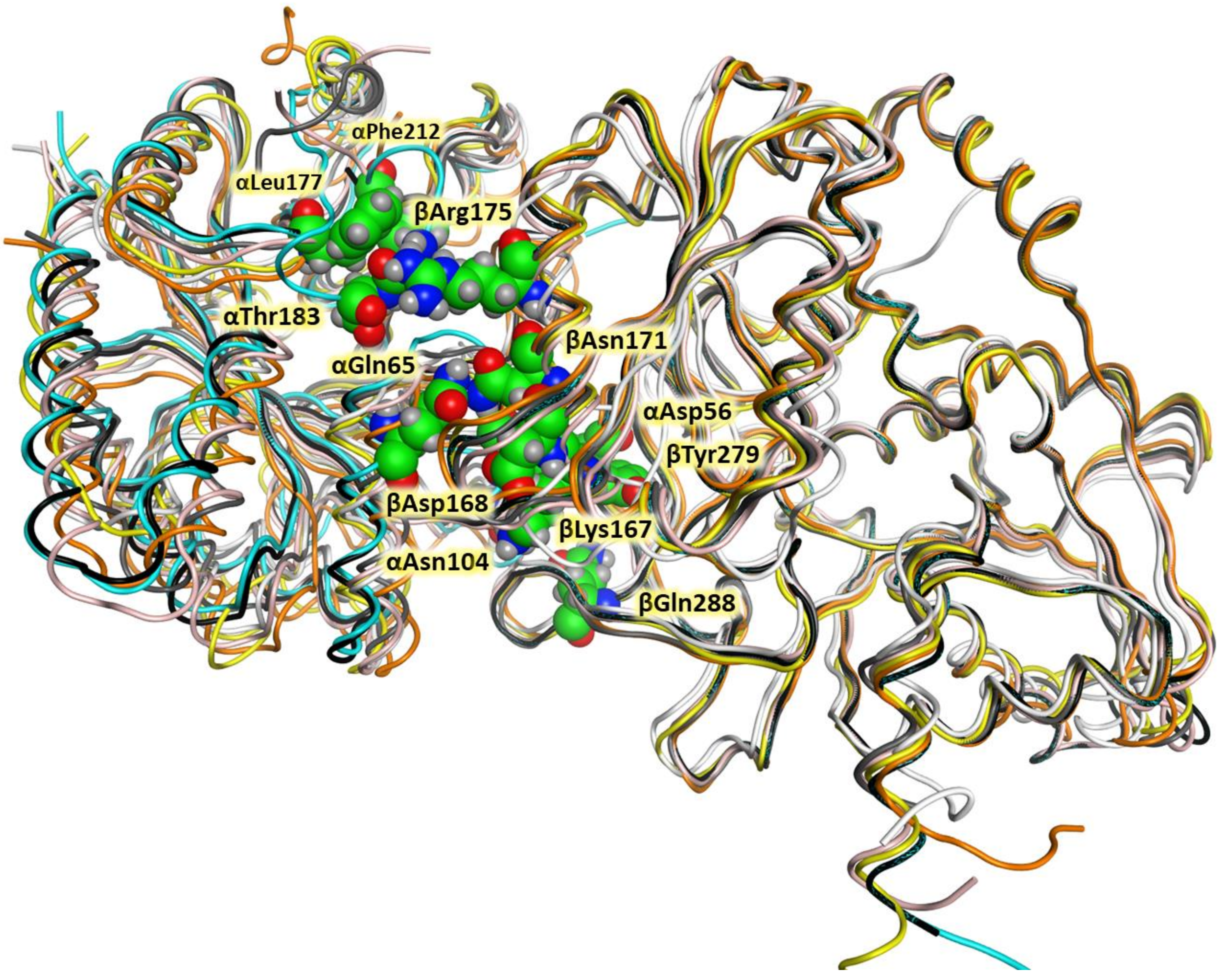

Supplementary Figure 14. Structural alignment of six structures of TS (PDB ID: 6DWE, 5KZM, 5KIN, 1WDW, 6V82, 1QOQ, color-coded as in Supplementary Figure 13) were aligned with our *E. coli* model. Highlighted residues are shown as VDW model. Conserved residues, showing conformational changes due to the A198W substitution are highlighted.
